# What is kangaroo mother care? Systematic review of the literature

**DOI:** 10.7189/jogh.06.010701

**Published:** 2016-06

**Authors:** Grace J Chan, Bina Valsangkar, Sandhya Kajeepeta, Ellen O Boundy, Stephen Wall

**Affiliations:** 1 Department of Pediatrics, Harvard Medical School, Boston, MA, USA; 2 Department of Global Health and Population, Harvard T.H. Chan School of Public Health, Boston, MA, USA; 3 Saving Newborn Lives, Save the Children, Washington, DC, USA

## Abstract

**Background:**

Kangaroo mother care (KMC), often defined as skin–to–skin contact between a mother and her newborn, frequent or exclusive breastfeeding, and early discharge from the hospital has been effective in reducing the risk of mortality among preterm and low birth weight infants. Research studies and program implementation of KMC have used various definitions.

**Objectives:**

To describe the current definitions of KMC in various settings, analyze the presence or absence of KMC components in each definition, and present a core definition of KMC based on common components that are present in KMC literature.

**Methods:**

We conducted a systematic review and searched PubMed, Embase, Scopus, Web of Science, and the World Health Organization Regional Databases for studies with key words “kangaroo mother care”, “kangaroo care” or “skin to skin care” from 1 January 1960 to 24 April 2014. Two independent reviewers screened articles and abstracted data.

**Findings:**

We screened 1035 articles and reports; 299 contained data on KMC and neonatal outcomes or qualitative information on KMC implementation. Eighty–eight of the studies (29%) did not define KMC. Two hundred and eleven studies (71%) included skin–to–skin contact (SSC) in their KMC definition, 49 (16%) included exclusive or nearly exclusive breastfeeding, 22 (7%) included early discharge criteria, and 36 (12%) included follow–up after discharge. One hundred and sixty–seven studies (56%) described the duration of SSC.

**Conclusions:**

There exists significant heterogeneity in the definition of KMC. A large number of studies did not report definitions of KMC. Skin–to–skin contact is the core component of KMC, whereas components such as breastfeeding, early discharge, and follow–up care are context specific. To implement KMC effectively development of a global standardized definition of KMC is needed.

Globally, 44% of under–five deaths occur during the neonatal period, and the proportion of under–five deaths due to neonatal causes continues to rise [1,2]. Preterm birth (before 37 weeks gestation) accounts for 35% of neonatal deaths. Low birth weight (defined as <2500 g) is commonly used as a surrogate measure of preterm birth [3]. Preterm and low birth weight infants who survive the neonatal period are more likely to experience neonatal morbidities including acute respiratory, gastrointestinal, immunologic, central nervous system, hearing and vision problems than both term and normal weight infants [4].

A significant proportion of deaths among preterm and low birth weight infants is preventable. There is evidence that kangaroo mother care (KMC), when compared to conventional neonatal care in resource–limited settings, significantly reduces the risk of mortality in infants born in facilities who are clinically stable and weighing less than 2000 g [5]. KMC also reduces the risk of hypothermia, severe illness, nosocomial infection, and length of hospital stay, and improves growth, breastfeeding, and maternal–infant attachment [5,6].

Despite strong evidence for mortality and morbidity reduction in low– and middle–income settings and endorsement from the World Health Organization (WHO), country–level adoption and implementation of KMC has been limited. In a systematic assessment of health system bottlenecks among countries with a high burden of neonatal deaths, KMC was identified as an intervention with significant health systems barriers to scale–up including leadership and governance, health financing, health workforce, health service delivery, health information systems, and community ownership and partnership [7]. Health intervention priority–setting tools, such as the Lives Saved Tool and Child Health and Nutrition Research Initiative methodology, have identified KMC as a high priority intervention based on criteria such as mortality benefit and equity [8,9].

In response to limited global uptake of KMC, in 2013, a group of newborn health stakeholders led by the Bill and Melinda Gates Foundation and Save the Children’s Saving Newborn Lives Program launched a global KMC Acceleration Convening. The goal was to address barriers to implementation, increase uptake of KMC as part of an integrated Reproductive Maternal Newborn and Child Health package, and identify research priorities [10]. In addition to implementation barriers, a lack of a clear definition of KMC has made *effective* coverage at scale of KMC challenging. A multi–country study in Africa found variation in KMC implementation across facilities in countries with national commitment to KMC [11]. Regional, country, and facility differences in health worker capacity, financial resources, leadership, health information systems, and cultural and community structures create challenges to developing and adopting a global definition of KMC.

The WHO has defined KMC as early, continuous, and prolonged skin–to–skin contact (SSC) between the mother and preterm babies; exclusive breastfeeding or breast milk feeding; early discharge after hospital–initiated KMC with continuation at home; and adequate support and follow–up for mothers at home [12]. While the WHO provides guidance on the components of KMC, guidance on the operationalization and clinical implementation of KMC are needed. There are significant variations in the timing of initiation, duration of SSC, positioning, necessary equipment and supplies, discharge criteria, follow–up frequency, indicators and measurement, and health workforce needs. The variations in these components have differential effects on preterm and low birth weight outcomes. As the global newborn health community begins to accelerate implementation of KMC, a standardized operational definition is needed. We conducted a systematic review of the KMC literature to 1) describe the current definitions of KMC in various settings, 2) analyze the presence or absence of WHO KMC components in each definition, and 3) present a core definition of KMC–common components that are present in at least 70% of all studies and programs–and describe how KMC definitions vary by context. This review provides a basis for development of an operational definition and clinical standards to accelerate the uptake of KMC globally.

## METHODS

We searched PubMed, Embase, Web of Science, Scopus, and WHO regional databases: AIM, LILACS, IMEMR, IMSEAR, and WPRIM using the search terms “kangaroo mother care”, “kangaroo care”, and “skin to skin care” with no language restrictions from 1 January 1960 to 24 April 2014 for original reports including case–control studies, cohort studies, randomized control trials, and case series with 10 or more participants (see **Online Supplementary Document(Online Supplementary Document)** for the review protocol and full search strategy). Following PRISMA guidelines, studies were included if they contained at least one of the following: the amount of time KMC was practiced, an association between KMC (as an isolated exposure, not part of a larger package) add any outcome, barriers to implementing KMC or factors necessary for successful implementation of KMC. Exclusion criteria were non–human subjects, case series or descriptive studies with fewer than 10 participants, and non–primary data collection or analysis (eg, reviews, meeting abstracts, editorials). Our population of interest included mothers, newborns, or mother–newborn dyads (not restricted to any specific ages) who have practiced KMC as well as health care providers, health facilities, communities, and health systems that have implemented KMC.

We also conducted hand–searches through the reference lists of the articles included in our review and published systematic reviews. Cochrane reviews were searched for relevant articles. To search the “grey literature” for unpublished studies, we explored programmatic reports and requested data from programs implementing KMC to obtain programmatic perspectives in addition to those provided by research studies. Reports were included following the same criteria as above.

Two independent reviewers examined titles, abstracts and full–text articles for inclusion into the review using a screening form based on our inclusion criteria. Using standardized data abstraction forms, two reviewers abstracted data independently from all included articles and reports. At each stage, reviewers compared results to ensure agreement. In the case of disagreement between the two reviewers, a third party acted as a tiebreaker. Native speakers abstracted data from articles in foreign languages. Languages for which a native speaker was not identified (ie, German, Finnish, Korean, Thai and Polish) were translated using an online translation software to assist with data abstraction. If an article or report were missing any information, we contacted the authors to request the data.

Using standardized forms, data were abstracted on study characteristics such as study design, country, sample size, location, and duration of follow–up. We abstracted data on KMC definitions including data on SSC, exclusive breastfeeding, early discharge from the facility, and follow–up and as well as other components [12]. We generated categorical variables for each component and calculated descriptive frequencies, means, medians and ranges for quantitative data.

## RESULTS

### Study selection and characteristics

**Figure 1:**
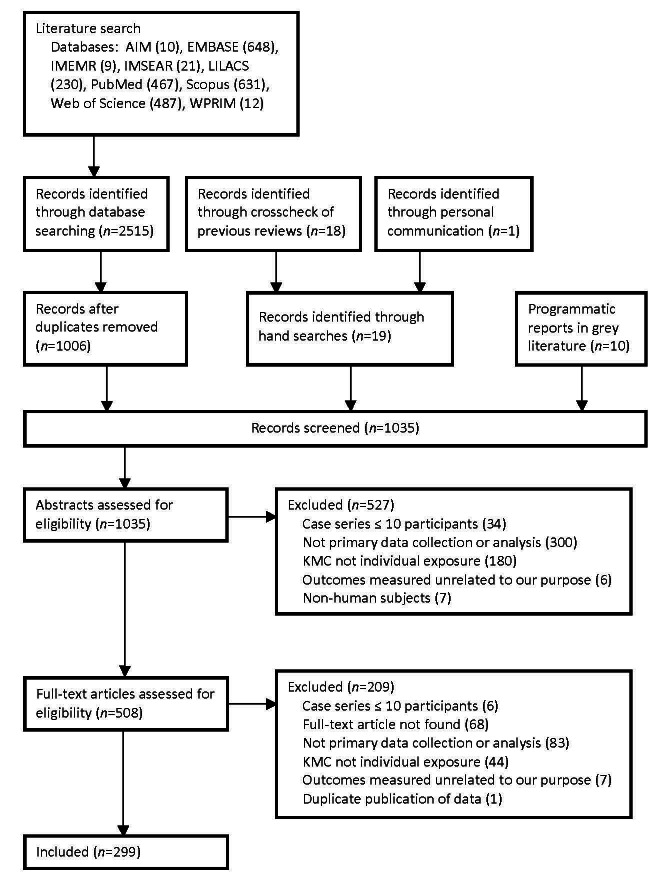
Flow diagram of study selection.

Our search strategy yielded 1035 records of which 299 were included in our review (**Figure 1**). Details of each included study are found in Table S1 in **Online Supplementary Document(Online Supplementary Document)**. Summary characteristics of the included studies are presented in **Table 1**. In the last five years, as KMC research gaps have gained growing attention, the number of studies conducted has increased. One hundred and thirty–four studies (45%) were published in the last five years between 2010 and 2014, 134 (45%) between 2000 and 2009, and 31 (10%) between 1988 and 1999. Common study types were randomized control trials (n = 85, 28%), surveys or interviews (n = 58, 19%), and cohorts (n = 43, 14%). Other study types included pre–post studies, facility–level evaluations, non–randomized intervention studies, and randomized crossover trials. One hundred and forty–four studies (48%) had less than 50 participants and 47 (16%) had 200 or more participants. Geographically, 115 (38%) of the studies took place in the Americas, 64 (21%) in Europe, 44 (15%) in Africa, 29 (10%) in Southeast Asia, 20 (7%) in Western Pacific, and 16 (5%) in Eastern Mediterranean regions. More studies were in countries with low neonatal mortality rates (NMRs), ie, less than 5 per 100 live births (n = 130, 43%), than in countries with high NMRs, ie, 30 or higher (n = 10, 3%) [13]. The majority of studies, 192 (64%), were in an urban setting. One hundred and seventy–five studies (59%) took place in health facilities, 107 (36%) in neonatal intensive care units or stepdown units, and 11 (4%) were community or population–based.

**Table 1:** Characteristics of included studies

	N = 299	%
**Year:**		
2010 to 2014	134	44.82
2000 to 2009	134	44.82
1988 to 1999	31	10.36
**Sample size:**		
<50	144	48.16
50 to <100	61	20.40
100 to <200	47	15.72
≥200	47	15.72
**Study type:**		
Randomized control trial	85	28.43
Surveys or interview	58	19.40
Cohort study	43	14.38
Pre–post intervention study	33	11.04
Facilities evaluation	23	7.69
Intervention trial	15	5.02
Randomized cross over	14	4.68
Other (chart review, case–control, cross over, surveillance)	28	9.36
**World Health Organization region:**		
Americas	115	38.46
Europe	64	21.40
Africa	44	14.72
Southeast Asia	29	9.70
Western Pacific	20	6.69
Eastern Mediterranean	16	5.35
Multiple regions	4	1.34
Missing	7	2.34
**Neonatal mortality rate (death per 1000 live birth):**		
<5	130	43.48
5 to <15	84	28.09
15 to <30	66	22.07
≥30	10	3.34
Missing	9	3.01
**Setting (rural or urban):**		
Urban	192	64.21
Urban and rural	23	7.69
Rural	10	3.34
Missing	74	24.75
**Population source:**		
Health facility	175	58.53
Neonatal intensive care unit or stepdown unit	107	35.79
Community or population–based surveillance	11	3.68
Missing	6	2.01
**Gestational age:**		
Preterm 34 to <37 weeks	57	19.06
Very preterm <34 weeks	51	17.06
Full term ≥37 weeks	33	11.04
Mixed preterm and very preterm <37 weeks	26	8.70
All gestational ages	28	9.36
Missing	104	34.78
**Birth weight:**		
Low birth weight 1500 to <2500 g	52	17.39
Mixed low <2500 g and very low birth weight <1500 g	45	15.05
All birth weights	25	8.36
Very low birth weight <1500 g	21	7.02
Non low birth weight ≥2500 g	9	3.01
Low birth weight vs non–low birth weight	1	0.33
Missing	146	48.83

Most studies included preterm newborns less than 37 weeks gestation (n = 134, 45%), 33 studies (11%) included only full term infants 37 weeks gestation or greater, 28 studies (9%) included newborns of all gestational ages, and 104 studies (35%) did not report gestational ages of the study participants. Similarly, 73 studies (24%) were among low birth weight infants less than 2500 g; 52 studies (17%) included infants less than 2500 g to 1500 g, and 21 (7%) studies were among very low birth weight infants less than 1500 g. Forty–five studies (15%) included a mix of low and very low birth weight newborns. Nine studies (3%) were among newborns weighing 2500 g or greater and 25 studies (8%) included newborns of all birth weights. One hundred forty–six studies (49%) did not describe birth weight characteristics. Forty three studies (14%) reported neither gestational age nor birth weight.

### KMC components

The individual components of KMC varied across studies (**Table 2**). Kangaroo mother care was not defined in 88 studies (29%). All 211 studies (71%) with KMC definitions included SSC as a component. One–hundred forty–eight studies (50%) included SSC only. For the additional components, 49 studies (16%) included SSC and exclusive or near–exclusive breastfeeding, 36 (12%) included SSC and follow–up after discharge from the health facility, and 22 (7%) included early discharge from the health facility.

**Table 2:** Description of kangaroo mother care components in studies

Kangaroo mother care components	N = 299	%
Skin–to–skin contact only	148	49.50
Skin–to–skin contact, breastfeeding	25	8.36
Skin–to–skin contact, breastfeeding, follow–up	16	5.35
Skin–to–skin contact, early discharge, follow–up	13	4.35
Skin–to–skin contact, breastfeeding, early discharge, follow up	7	2.34
Skin–to–skin contact, breastfeeding, early discharge	1	0.33
Skin–to–skin contact, early discharge	1	0.33
Undefined kangaroo mother care	88	29.43

### Skin–to–skin contact

Among the studies that defined SSC as part of the KMC package, criteria for SSC initiation, SSC ending, and SSC duration were not well described (**Table 3** and **Table 4**). In 43 studies (14%), SSC was initiated after non–stability criteria were met, 27 studies (9%) promoted immediate initiation of SSC within 60 minutes of birth, 76 studies (25%) encouraged SSC after stability criteria were met, 18 studies (6%) encouraged SSC after a painful procedure, and 135 (45%) did not describe SSC initiation criteria. Forty–three studies observed initiation of SSC of which 4 (9%) observed immediate initiation of SSC. Criteria for stability were non–specific including the terms “clinically stable,” “adapted to extra–uterine life,” “can tolerate handling,” and “without serious illness”. More defined criteria included “satisfactory APGAR score,” “stable weight,” and “stable respiratory and hemodynamic parameters.” Criteria to end SSC were largely non–specific with terms “one day or less,” “until baby no longer accepts,” or “until parent no longer accepts.” More specific terms included “until reaches satisfactory weight [2000 grams or 2500 grams]”. We compared descriptions of SSC with observations of SSC to differentiate promotion vs practice. Most studies (>85%) did not include data on observations of SSC practiced (**Table 3**).

**Table 3:** Promoted skin–to–skin contact characteristics compared to observed skin–to–skin contact characteristics

	Promoted skin–to–skin contact		Observed skin–to––skin contact	
**N**	**%**	**N**	**%**
**Skin–to–skin contact initiation:**				
After stability criteria were met	76	25.42	11	3.68
After non–stability criteria were met	43	14.38	28	9.36
Immediately, regardless of stability	27	9.03	4	1.34
Prior to painful procedure	18	6.02	0	0.00
Undefined or not applicable	135	45.15	256	85.62
**Skin–to–skin contact stability criteria:**				
Respiratory and/or hemodynamically stable	28	9.36	2	0.67
Clinically stable–not specified further	20	6.69	5	1.67
Adapted to extra–uterine life	8	2.68	0	0.00
Without serious illness	7	2.34	2	0.67
Can tolerate handling	6	2.01	1	0.33
Stable weight	4	1.34	1	0.33
Satisfactory APGAR score	2	0.67	0	0.00
Term	1	0.33	0	0.00
Undefined or not applicable	223	74.58	288	96.32
**When was skin–to–skin contact instructed to end?**				
One day or less	48	16.05	5	1.67
Until baby no longer accepts	22	7.36	1	0.33
Shortly after painful procedure	13	4.35	0	0.00
After one day and up to two weeks	11	3.68	5	1.67
Until reaches satisfactory weight (2000; 3000 g)	10	3.34	5	1.67
After two weeks	8	2.68	5	1.67
Until parent or baby no longer accepts	7	2.34	0	0.00
Until discharge	4	1.34	3.00	1.00
Until parent no longer accepts	4	1.34	0	0.00
Until reached satisfactory health status	3	1.00	0	0.00
Undefined or not applicable	169	56.52	275	91.97

**Table 4:** Promoted skin–to–skin contact duration compared to observed skin–to–skin contact duration

	Promoted skin–to–skin contact duration		Observed skin–to–skin contact duration	
**N = 299**	**%**	**N = 299**	**%**
**Skin–to–skin contact continuous or intermittent within session:**				
Continuous within one session	117	39.13	16	5.35
Continuous (24 h per day)	44	14.72	7	2.34
Intermittent (multiple sessions)	26	8.70	17	5.69
Undefined or not applicable	112	37.46	259	86.62
**Skin–to–skin contact duration (hours per session):**				
1 to 2 sessions	90	30.10	13	4.35
3 to 4 sessions	11	3.68	0	0.00
5 to 8 sessions	2	0.67	0	0.00
≥8 sessions	0	0.00	1	0.33
Undefined or not applicable	196	65.55	285	95.32
**Skin–to–skin contact duration (number hours per day):**				
<2 h	78	26.09	13	4.35
2 to <4 h	28	9.36	3	1.00
4 to <9 h	13	4.35	8	2.68
9 to <12 h	1	0.33	3	1.00
12 to <22 h	1	0.33	4	1.34
≥22 h	46	15.38	6	2.01
Undefined or not applicable	132	44.15	262	87.63
**Skin–to–skin contact duration (number days):**				
1 to 5 d	74	24.75	11	3.68
6 to <30 d	19	6.35	8	2.68
≥30 d	5	1.67	1	0.33
Dependent on hospital stay	7	2.34	1	0.33
Undefined or not applicable	194	64.88	278	92.98

Data on the duration of SSC are needed to understand the benefits of SSC as well as the feasibility to scale KMC; however this was missing from most studies (**Table 4**). One hundred thirty–two studies (44%) did not describe the number of hours per day SSC was promoted. Seventy–eight studies (26%) encouraged SSC for less than two hours per day, 15 of these studies examined the effect of SSC on painful procedures. Otherwise, the most common duration of SSC promoted was 22 hours or more (n = 46, 15%). Only 37 studies (12%) observed duration of SSC practiced, of which six (2%) observed at least 22 hours per day SSC practiced. SSC duration was also categorized inconsistently as continuous, intermittent, number of hours per session, number of sessions per day, and number of days. Definitions of the term continuous included 24 hours per day, continuous within sessions, or one continuous session but less than 24 hours a day.

### Breastfeeding

Breastfeeding habits were reported in 105 (35%) studies: 38 (13%) reported exclusive breastfeeding, 22 (7%) nearly–exclusive breastfeeding, and 35 (12%) breastfeeding and supplemental feeding (**Table 5**). In most studies, breastfeeding initiation time was not reported (n = 261, 87%). Breastfeeding was started immediately or within one hour of birth in 15 studies (5%), between one and 24 hours after birth in two studies (1%), and 24 hours or longer after birth in five studies (2%). In nine studies (3%) breastfeeding was started at KMC initiation, and seven studies (2%) included physical maturity criteria for initiation of breastfeeding. Seventeen studies (6%) described breastfeeding frequency in their patient population, 13 (4%) studies reported women breastfeeding every two to three hours and four studies (1%) reported women breastfeeding whenever possible.

**Table 5:** Description of breastfeeding characteristics

	N = 299	%
**Breastfeeding habits:**		
Exclusive	38	12.71
Mixed with other food	35	11.71
Nearly exclusive	22	7.36
Combination	8	2.68
No breastfeeding	2	0.67
Undefined or not applicable	194	64.88
**When did breastfeeding start?:**		
Immediately or within one hour of delivery	15	5.02
When kangaroo mother care started	9	3.01
Once reached satisfactory degree of physical maturity	7	2.34
One day or more after birth	5	1.67
After one hour but within 24 h of birth	2	0.67
Undefined or not applicable	261	87.29
**Breastfeeding frequency:**		
Every two to three hours	13	4.35
Whenever possible	4	1.34
Undefined or not applicable	282	94.31

### Discharge criteria from facility

Fourteen percent of studies (n = 42) described the criteria used for hospital discharge in their study populations (**Table 6**). The most common criteria were clinical stability (n = 19, 6%) or meeting a specified weight gain or weight minimum cutoff (n = 15, 5%). Seven studies (2%) required a combination of adequate weight gain and exclusive breastfeeding prior to discharge. Most studies did not report when infants were discharged (n = 285, 95%). Six studies (2%) reported discharge within seven days of life and eight studies (3%) reported discharge after seven days of life.

**Table 6:** Description of discharge and follow–up characteristics

	N = 299	%
**Discharge criteria:**		
Clinically stable	19	6.35
Adequate weight gain	10	3.34
Exclusively breastfeeding and consistently gaining weight	7	2.34
Absolute weight cutoff	5	1.67
Neonatologist approval	1	0.33
Within time of birth	0	0.00
Undefined or not applicable	257	85.95
**Discharge timing:**		
After seven days of life	8	2.68
Within seven days of life	6	2.01
Undefined or not applicable	285	95.32
**Follow–up location:**		
Facility	29	9.70
Home	22	7.36
Facility and home	9	3.01
Phone call or letter	1	0.33
Undefined or not applicable	238	79.60
**Follow–up time:**		
>3 months to 6 months	11	3.68
>6 months to 12 months	11	3.68
Dependent on adequate weight gain	10	3.34
≤1 months	8	2.68
>1 months to 3 months	8	2.68
Until 40 weeks gestational age	4	1.34
>12 months to 18 months	2	0.67
Undefined or not applicable	245	81.49
**Compliance with follow–up:**		
70 to <90%	11	3.68
90 to <100%	9	3.01
<70%	7	2.34
100%	2	0.67
Undefined or not applicable	270	90.30

### Follow–up

Sixty–one studies (20%) described follow–up of infants after discharge, of which 29 studies (48%) followed–up with newborns in health facilities, 22 studies (36%) in homes, and 9 studies (15%) in both facilities and homes (**Table 6**). Follow–up time varied from one month or less (n = 8, 3%) to six to 18 months (n = 13, 4%). Most studies (n = 270, 90%) did not report compliance with follow–up, 11 (4%) reported 90% or higher compliance.

### Other components

Studies also described clothing recommendations, newborn positioning, and temperature monitoring during KMC. In 64 studies (21%) participants were instructed to clothe their infant in only a diaper during kangaroo care, an additional 64 studies (21%) encouraged use of a diaper, cap, and socks, and 17 (6%) promoted having the infant naked during SSC contact (**Table 7**). The majority of studies (n = 179, 60%) instructed participants to position the infant prone on the care provider’s chest during SSC, while five studies (2%) encouraged a side–lying or breastfeeding position. In 59 studies (20%), the kangaroo care provider was instructed to be in a reclined position, while an upright position was encouraged in 48 studies (16%). Temperature of the infant was monitored during SSC in 71 studies (24%).

**Table 7:** Description of clothing and positioning during kangaroo mother care

	Promoted clothing and positioning		Observed clothing and positioning			
**N**	**%**	**N**	**%**
**Clothing of kangarooed baby:**				
Diaper or nappy	64	21.40	8	2.68
Diaper, cap, and socks	64	21.40	6	2.01
Naked	17	5.69	2	0.67
Undefined or not applicable	154	51.51	283	94.65
**Position of kangarooed baby:**				
Prone on mother's chest	179	59.87	17	5.69
On side or next to mother	3	1.00	1	0.33
Breastfeeding position	2	0.67	0	0.00
Undefined or not applicable	115	38.46	281	93.98
**Position of provider:**				
Inclined or reclined	59	19.73	8	2.68
Upright	48	16.05	5	1.67
Variation of inclined and upright	12	4.01	2	0.67
Undefined or not applicable	180	60.20	284	94.98

## DISCUSSION

There is significant heterogeneity in the definition of KMC and a large number of studies did not report a definition of KMC. Of the studies that defined KMC, SSC was present in all studies. Additional KMC components – breastfeeding, early discharge, and follow–up–were missing in the majority of studies. These findings suggest that SSC is accepted in research and programmatic settings as an essential component of KMC, but the other components vary by context, defined as demographic, economic, social, and cultural factors, and newborn characteristics.

The lack of a clear KMC definition and guidance for implementing KMC is a reflection of incomplete evidence. Evidence for KMC is largely based on meta–analyses that combine studies with heterogeneous definitions of KMC and occur in different settings [5,6]. Attempts to stratify the association of KMC on outcomes by KMC components, newborn characteristics (birth weight, gestational age), and high NMR vs low NMR often do not yield statistically significant results because of the limited data available. We do not know the effect of different combinations of KMC components, nor do we understand the feasibility with which each component can be implemented effectively in different contexts. Our study was limited by the lack of data on the duration of SSC. Furthermore, measurement of SSC duration was based on mothers’ report of time with minimal observational data. Studies where SSC duration was measured by an independent observer may be biased by the Hawthorn effect.

To define the optimal duration of SSC, we need additional data on the dose response of SSC duration on mortality and morbidity outcomes. The benefits of SSC are likely dependent on the duration of SSC, however the duration of SSC must also be balanced with the feasibility of practicing SSC for extended periods of time. In most settings promoting SSC 24 hours a day is not feasible. Understanding the minimal duration of SSC that provides the maximal benefits will provide more specific recommendations. Most studies initiated KMC after stabilization of the newborn and the effect of KMC on mortality and morbidity is generalizable to the population of newborns who survive to be stabilized. The effect of KMC immediately after birth before stabilization is unclear due to inconclusive evidence [14–17]. Additional efforts to test the effect of KMC prior to stabilization and to define stability is needed through further studies or by consulting experts at each level of care (primary, secondary, or tertiary care) through a Delphi method.

To operationalize KMC, the simpler the intervention the more likely it is to scale [18]. A simple and clear operational definition for KMC is needed. Evidence suggests benefits for newborns less than 2000 g, who are stabilized in facilities with SSC as the primary component. More work is needed to improve the measurement of gestational age and improving the recording of birth weights in facilities to better understand the impact of KMC and for whom there are benefits. Our review suggests that skin–to–skin contact is the core minimal component of KMC and variations depend on context and individual clinical needs of the newborn. For example, extremely preterm newborns who are unable to coordinate their suck and swallow will need feeding support such as nasogastric feeding or intravenous fluid. In high resource settings with space and infection precautions, a provider may recommend SSC for a preterm infant but choose not to discharge early from the facility. To operationalize KMC, a simple matrix that lists newborn characteristics in columns and KMC components in rows for different settings, ie, tertiary, secondary, primary or community levels, can take into account the core SSC components with variations based on differences in the newborn and context.

As implementation of KMC begins to accelerate globally, data on the context, individual newborn factors, and KMC components can be collected and harmonized to generate a model that will best define KMC for a set of individual newborn characteristics in specific settings. Research and programmatic agendas to advance KMC should include a standardized set of indicators and measurement tools that document SSC initiation criteria, SSC duration as number of hours per day promoted and ideally observed, feeding protocols, discharge criteria from a facility to community and follow–up standards, and discharge criteria from KMC. To track progress, indicators and standard measurement tools are needed to measure coverage of key newborn interventions including KMC [19]. The release of the new preterm guidelines by the World Health Organization, where KMC is recommended for all newborns less than 2000 g, will provide an opportunity for programs and researchers to start addressing definition gaps, establish global recommendations of operational definitions and core components of KMC, and accelerate KMC within care of preterm babies.

### CONCLUSION

Developing a standardized operational definition of KMC and employing indicators and measurement tools to measure and evaluate KMC acceleration efforts is needed. More than half of the studies equate KMC with SSC. Moving forward, careful distinction between KMC and SSC is needed. While SSC is beneficial for all newborns, KMC should be clearly defined, at the bare minimum, as a package of interventions including SSC, exclusive breastfeeding, and close monitoring for preterm and/or low birthweight babies. Researchers and program implementers can contribute to building a more solid evidence base for KMC by measuring and reporting how KMC is defined–the components implemented and the feasibility of implementation based on the context–and the outcomes measured. A central and accessible database to share knowledge should contain this data in addition to standardized indicators, such as the proportion of eligible newborns who receive KMC and the barriers and facilitators to implementation of KMC.
