# Supplementary material for: What is kangaroo mother care? Systematic review of the literature
Source: J Glob Health. 2016 Mar 15;6(1):010701. doi: 10.7189/jogh.06.010701 (PMC4871067; doi:10.7189/jogh.06.010701)
Supplement: Online Supplementary Document [file jogh-06-010701-s001.pdf]

Online Supplementary Document

Chan et al. What is kangaroo mother care? Systematic review of the literature

J Glob Health 2016;6:010701

Table S1. Studies included in the kangaroo mother care definition systematic review (N=299), 2014

Author	Year	Title	Country	Study design	Sample size	Newborns Characteristics	KMC components	Onset of SSC	Stability criteria for KMC initiation	Hours per day of KMC provision*	# of days of KMC provision*	Discharge criteria for Kangarooed baby	Follow-up duration	Follow-up location
Abouelfettoh	2011	Effect of skin-to-skin contact on preterm infant skin barrier function and hospital-acquired infection	USA	Pre-post	10 Dyads	28-30 weeks	SSC	N/A	N/A	≥1	5	N/A	N/A	N/A
Abul-Fadl	2012	Evaluation of mothers' knowledge, attitudes, and practice towards the ten steps to successful breastfeeding in Egypt	Egypt	Pop based surveillance, facility evaluation	1052 Mothers	All ages	SSC	N/A	N/A	N/A	N/A	N/A	N/A	N/A
Abul-Fadl	2011	Breastfeeding and skin-to-skin care in congenital heart disease with heart failure	Egypt	Focus group/ interview	1052 Mothers	<24 mon.	N/A	N/A	N/A	N/A	N/A	N/A	N/A	N/A
Acharya	2014	Randomized control trial of Kangaroo Mother Care in low birth weight babies at a tertiary level hospital	Nepal	RCT	126 Newborns	<2000g	N/A	Once both baby and mother were ready	N/A	≥6	N/A	N/A	N/A	N/A

Author	Year	Title	Country	Study design	Sample size	Newborns Characteristics	KMC components	Onset of SSC	Stability criteria for KMC initiation	Hours per day of KMC provision*	# of days of KMC provision*	Discharge criteria for Kangarooed baby	Follow-up duration	Follow-up location
Acosta Díaz	2003	Kangaroo mother care method. Clinical and humoral assessment during the first year of corrected age	Cuba	Intervention	120 Newborns	Premature: N/A cutoff	SSC	N/A	N/A	N/A	N/A	N/A	At 3 and 6 mon. GA	N/A
Acosta Díaz	2003	Kangaroo mother care method. Evaluation of the neurodevelopment at first year of corrected age	Cuba	Intervention	120 Newborns	Premature: N/A cutoff	SSC	N/A	N/A	N/A	N/A	N/A	At 3 and 6 mon. GA	N/A
Acosta Díaz	2003	Kangaroo mother care method. Impact on the physical and intellectual development at preschool age	Cuba	Retrospective cohort	100 Newborns	Premature: N/A cutoff	SSC	Immediately after birth	N/A	N/A	N/A	N/A	N/A	N/A
Acuna-Muga	2013	Volume of milk obtained in relation to location and circumstances of expression in mothers of very low birth weight infants	Spain	Intervention	26 Dyads	VLBW: N/A cutoff	N/A	After mother was eligible	N/A	≥2	≥5	N/A	N/A	N/A
Aghdas	2014	Effect of immediate and continuous mother-infant skin-to-skin contact on breastfeeding self-efficacy of primi-parous	Iran	RCT	92 Mothers	≥37 weeks, 2500-4000g	SSC	Once eligible: N/A definition	N/A	≥2	N/A	N/A	N/A	N/A

Author	Year	Title	Country	Study design	Sample size	Newborns Characteristics	KMC components	Onset of SSC	Stability criteria for KMC initiation	Hours per day of KMC provision*	# of days of KMC provision*	Discharge criteria for Kangarooed baby	Follow-up duration	Follow-up location
		women: A randomized control trial												
Ahmed	2011	Community Kangaroo Mother Care: implementation and potential for neonatal survival and health in very low-income settings	Bangladesh	Prospective cohort	4165 Dyads	All ages	SSC, EBF, FU	N/A	N/A	N/A	N/A	N/A	N/A	Home
Ahn	2010	Kangaroo care on premature infant growth and maternal attachment and post-partum depression in South Korea	South Korea	Prospective Cohort	20 Dyads	<36 weeks, <1800g	N/A	N/A	N/A	≥1	21	N/A	N/A	N/A
Akcan	2009	The effect of kangaroo care on pain in premature infants during invasive procedures	Turkey	RCT	50 Newborns	26-36 PMA, LBW	SSC	N/A	N/A	0.75	5	N/A	N/A	N/A
Ali	2009	Kangaroo mother care as compared to conventional care for low birth weight babies	India	RCT	114 Newborns	1200-1800g	SSC	As soon as baby hemodynamically became stable	The same as SSC onset criteria	≥4-6	N/A	Weight gain for at least 3 consecutive days, no overt illness, no IV medications and on exclusive breast feeds	N/A	N/A

Author	Year	Title	Country	Study design	Sample size	Newborns Characteristics	KMC components	Onset of SSC	Stability criteria for KMC initiation	Hours per day of KMC provision*	# of days of KMC provision*	Discharge criteria for Kangarooed baby	Follow-up duration	Follow-up location
Almeida	2007	Effects of Kangaroo Mother Care on the vital signs of low-weight preterm newborns	Brazil	Pre-post	22 Newborns	28-33 weeks	SSC	After first assessment	N/A	0.5	3	N/A	N/A	N/A
Almeida	2010	The impact of kangaroo care on exclusive breastfeeding in low birth weight newborns	Brazil	Retrospective cohort	43 Newborns	<2000g	SSC, EBF	N/A	N/A	N/A	N/A	N/A	N/A	N/A
Alves	2007	Early weaning in premature babies participants of the Kangaroo Mother Care	Brazil	Chart review, Focus group/ interview	33 Dyads	Premature: N/A cutoff	N/A	As soon as possible	N/A	N/A	N/A	N/A	5 mon.	Health facility
Anderson	1986	Kangaroo care for premature infants	Colombia	Pop based surveillance	N/A Newborns	Premature: N/A cutoff	SSC, DC, FU	N/A	N/A	N/A	N/A	N/A, but after mother education about how to feed and perform KMC.	N/A	Health facility
Anderson	2003	Mother-newborn contact in a randomized trial of kangaroo (skin-to-skin) care	USA	RCT	91 Dyads	32-36 weeks, 1300-3000g	SSC, DC	Immediately after birth	N/A	24	2-5	If healthy enough to leave at 48 hours	N/A	N/A
Andrade	2005	Suckling of the premature newborn child: comparison between the kangaroo mother method with traditional care	Brazil	Prospective cohort, descriptive	30 Dyads	30-35 weeks	N/A	N/A	N/A	N/A	N/A	N/A	N/A	N/A

Author	Year	Title	Country	Study design	Sample size	Newborns Characteristics	KMC components	Onset of SSC	Stability criteria for KMC initiation	Hours per day of KMC provision*	# of days of KMC provision*	Discharge criteria for Kangarooed baby	Follow-up duration	Follow-up location
Araújo	2010	Mother Kangaroo Method: an investigation about the domestic practice	Brazil	Focus group/ interview	30 Parents	Premature: N/A cutoff, ≤2000g	N/A	Once eligible: N/A definition	N/A	5-6	N/A	≥1500 g, weight gain for 3 days , efficient suction and (EBF, and the mother motivated to continue KMC full-time, family support ,outpatient ensured and guaranteed if any abnormality occurring	3 times in the first week after discharge, 2 times in the second, once a week until reach 2500g	Home
Arivabene	2010	Kangaroo mother method: Mothers' experiences and contributions to nursing	Brazil	Focus group/ interview	13 Mothers	N/A	SSC	N/A	N/A	N/A	N/A	N/A	3rd phase of the study, between 2000-2006	Home
Arzani	2012	Kangaroo care effect on self-esteem in the mothers of low birth weight infants	Iran	RCT, pre/post	60 Mothers	1000-2500g	SSC	N/A	Physiological stability of infants was an inclusion criteria	≥2	N/A	N/A	N/A	N/A
Azevedo	2012	Safety of Kangaroo Mother Care in intubated neonates under 1500 g	Brazil	Pre-post	43 Newborns	Premature: N/A cutoff, <1500g	SSC	N/A	Respiratory and hemodynamically stable, no signs of pain observed	1	1	N/A	N/A	N/A
Azevedo	2011	Kangaroo mother care in preterm newborns on artificial ventilation: An evaluation of behavior patterns	Brazil	Pre-post	44 Newborns	29 weeks, 1096g average	N/A	N/A	N/A	N/A	N/A	N/A	N/A	N/A

Author	Year	Title	Country	Study design	Sample size	Newborns Characteristics	KMC components	Onset of SSC	Stability criteria for KMC initiation	Hours per day of KMC provision*	# of days of KMC provision*	Discharge criteria for Kangarooed baby	Follow-up duration	Follow-up location
Bang	2011	Perception of Nurses and Physicians in Neonatal Intensive Care Units on Kangaroo Care	South Korea	Focus group/ interview	149 Nurses, 19 Doctors	N/A	N/A	N/A	N/A	N/A	N/A	N/A	N/A	N/A
Bauer	1997	Body temperatures and oxygen consumption during skin-to-skin (kangaroo) care in stable preterm infants weighing less than 1500 grams	Germany	Pre-post	22 Newborns	<1500g	SSC	At 5pm after monitored in incubator and fed	N/A	1	7	N/A	N/A	N/A
Bauer	1996	Metabolic rate and energy balance in very low birth weight infants during kangaroo holding by their mothers and fathers	N/A	Pre-post	11 Newborns	28-32 weeks, 560-1390g	SSC	8-48 days after birth	N/A	1	1	N/A	N/A	N/A
Bazzano	2012	Introducing home based skin-to-skin care for low birth weight newborns: a pilot approach to education and counseling in Ghana	Ghana	Focus group/ interview	9 Mothers, 23 HCWs	LBW: N/A cutoff	SSC	N/A	N/A	N/A	N/A	N/A	N/A	N/A

Author	Year	Title	Country	Study design	Sample size	Newborns Characteristics	KMC components	Onset of SSC	Stability criteria for KMC initiation	Hours per day of KMC provision*	# of days of KMC provision*	Discharge criteria for Kangarooed baby	Follow-up duration	Follow-up location
Begum	2008	Cerebral oxygenation responses during kangaroo care in low birth weight infants	N/A	Pre-post	16 Newborns	<33 weeks, <1600g	SSC	After 30 minutes in incubator	N/A	1	1	N/A	N/A	N/A
Bera	2014	Effect of kangaroo mother care on growth and development of low birth weight babies up to 12 month of age: a controlled clinical trial	India	Intervention	500 Dyads	<2500g	SSC, EBF, FU	Once hemodynamically stable	The same as SSC onset criteria	1 on first day, 2 on second, 3 on third , and then as long as possible	N/A	N/A	N/A	Health facility
Bergh	2013	Progress with the implementation of kangaroo mother care in four regions in Ghana	Ghana	Facility evaluation	38 Facilities	N/A	SSC, EBF, FU	Immediately after birth	N/A	N/A	N/A	N/A	N/A	N/A
Bergh	2003	Development of a conceptual tool for the implementation of kangaroo mother care	South Africa	Facility evaluation	2 Facilities	N/A	N/A	N/A	N/A	N/A	N/A	N/A	N/A	N/A
Bergh	2012	Progress in the implementation of kangaroo mother care in 10 hospitals in Indonesia	Indonesia	Facility evaluation	10 Facilities	N/A	N/A	N/A	N/A	N/A	N/A	N/A	N/A	N/A

Author	Year	Title	Country	Study design	Sample size	Newborns Characteristics	KMC components	Onset of SSC	Stability criteria for KMC initiation	Hours per day of KMC provision*	# of days of KMC provision*	Discharge criteria for Kangarooed baby	Follow-up duration	Follow-up location
Bergh	2008	Scaling up kangaroo mother care in South Africa: 'on-site' versus 'off-site' educational facilitation	South Africa	RCT	36 Facilities	N/A	N/A	N/A	N/A	N/A	N/A	N/A	N/A	N/A
Bergh	2012	Translating research findings into practice--the implementation of kangaroo mother care in Ghana	Ghana	Pop based surveillance, facility evaluation	38 Facilities	N/A	N/A	N/A	N/A	N/A	N/A	N/A	N/A	N/A
Bergh**	2009	Translating research findings into policy and practice. The scaling up of kangaroo mother care in Ghana.	Ghana	Facility evaluation	38 Facilities	N/A	N/A	N/A	N/A	N/A	N/A	N/A	N/A	N/A
Bergh**	2009	Scaling up Kangaroo Mother Care in Ghana	Ghana	Facility evaluation	4 Regions (out of 10)	N/A	N/A	N/A	N/A	N/A	N/A	N/A	N/A	N/A
Bergh**	2012	Evaluation OF Kangaroo Mother Care Services in Malawi	Malawi	Facility evaluation	14 Facilities	N/A	N/A	N/A	N/A	N/A	N/A	N/A	until reached 2500g	Home and health facility
Bergh**	2012	Evaluation OF Kangaroo Mother Care Services in Mali	Mali	Facility evaluation	7 Facilities	N/A	SSC, EBF,DC, FU	N/A	N/A	N/A	N/A	EBF	Different for each hospital	Health facility
Bergh**	2007	Retrospective Evaluation of Kangaroo Mother Care Practices in Malawian Hospitals	Malawi, South Africa	Facility evaluation	6 Facilities	N/A	N/A	N/A	N/A	N/A	N/A	N/A	N/A	N/A

Author	Year	Title	Country	Study design	Sample size	Newborns Characteristics	KMC components	Onset of SSC	Stability criteria for KMC initiation	Hours per day of KMC provision*	# of days of KMC provision*	Discharge criteria for Kangarooed baby	Follow-up duration	Follow-up location
Bergh**	2012	Evaluation OF Kangaroo Mother Care Services in Rwanda	Rwanda	Facility evaluation	7 Facilities	N/A	N/A	N/A	N/A	N/A	N/A	N/A	N/A	N/A
Bergh**	2012	Evaluation OF Kangaroo Mother Care Services in Uganda	Uganda	Facility evaluation	11 Facilities	N/A	N/A	N/A	N/A	N/A	N/A	N/A	N/A	N/A
Bergman	2004	Randomized controlled trial of skin-to-skin contact from birth versus conventional incubator for physiological stabilization in 1200- to 2199-gram newborns	South Africa	RCT	35 Dyads	1200-2199g	SSC	After infant dried and assessed for anomalies, resuscitated if needed, and given eye prophylaxis and vit K injection and weighed	No need to resuscitate	6	1	N/A	N/A	N/A
Bergman	1994	The 'kangaroo-method' for treating low birth weight babies in a developing country	Mozambique	Chart review	126 Newborns	<2000g	SSC, EBF	After drying	N/A	N/A	N/A	At 2500 g and breast fed only	N/A	N/A
Bergstrom	2007	Immediate maternal thermal response to skin-to-skin care of newborn	Uganda	Pre-post	39 Dyads	Non LBW	SSC	Immediately after birth	N/A	0.5-1	1	N/A	N/A	N/A
Bier	1996	Comparison of skin-to-skin contact with standard contact in low-birth-weight in-	Colombia	RCT	50/ Dyads	<1500g	SSC, EBF	N/A	N/A	N/A	≤10	N/A	N/A	N/A

Author	Year	Title	Country	Study design	Sample size	Newborns Characteristics	KMC components	Onset of SSC	Stability criteria for KMC initiation	Hours per day of KMC provision*	# of days of KMC provision*	Discharge criteria for Kangarooed baby	Follow-up duration	Follow-up location
		fants who are breastfed												
Bigelow	2012	Effect of Mother/Infant Skin-to-Skin Contact on Postpartum Depressive Symptoms and Maternal Physiological Stress	Canada	Intervention	90 Mothers	Full term	SSC	N/A	N/A	6 in first week, 2 until 1 mon. of age	30	N/A	N/A	N/A
Bigelow	2010	The relation between early mother-infant skin-to-skin contact and later maternal sensitivity in South African mothers of low birth weight infants	South Africa	RCT, chart review, Focus group/ interview	12 Dyads	1385-2199	SSC	Immediately after birth	N/A	N/A	N/A	N/A	N/A	Home
Blay**	2009	Ghana KMC Sudy Results	Ghana	Chart review	12953 Newborns	All ages, 1000-2000g	N/A	N/A	N/A	N/A	N/A	N/A	N/A	N/A
Blencowe	2009	Safety, effectiveness and barriers to follow-up using an 'early discharge' Kangaroo Care policy in a resource poor setting	Malawi	Prospective Cohort	272 Newborns	<2000g	N/A	Once eligible: N/A definition	After initial stabilization in nursery	N/A	N/A	Weight >1300g and gaining weight for 3 days, maintain stable temperature (36-37.5 degrees C), able to breastfeed or cup feed expressed breast milk	Fortnightly until weight 2000g, then 3-weekly until 2500g	Home and health facility

Author	Year	Title	Country	Study design	Sample size	Newborns Characteristics	KMC components	Onset of SSC	Stability criteria for KMC initiation	Hours per day of KMC provision*	# of days of KMC provision*	Discharge criteria for Kangarooed baby	Follow-up duration	Follow-up location
Blencowe	2005	Setting up Kangaroo Mother Care at Queen Elizabeth Hospital, Blantyre - A practical approach	Malawi	Facility evaluation	1 Facility	<2000g	SSC, EBF, DC, FU	N/A	Stable, non-sick	N/A	N/A	Established breast or cup feeding , free of serious illness, weight > 1500g	N/A	Health facility
Blomqvist	2013	Initiation and extent of skin-to-skin care at two Swedish neonatal intensive care units	Sweden	Focus group/ interview	104 Newborns	27-33 weeks	SSC, DC, FU	N/A	N/A	N/A	N/A	N/A	At 34-35 weeks PMA	N/A
Blomqvist	2013	Provision of Kangaroo Mother Care: supportive factors and barriers perceived by parents	Sweden	Focus group/ interview	76 Mothers, 74 Fathers	28-33 weeks, 740-2920g	SSC	N/A	Without any life threatening illnesses	N/A	N/A	N/A	1-2 weeks after discharge	Home by letter
Blomqvist	2011	Swedish mothers' experience of continuous Kangaroo Mother Care	Sweden	Focus group/ interview	23 Dyads	All ages	SSC, EBF	N/A	N/A	N/A	N/A	N/A	N/A	N/A
Blomqvist	2010	Parent-infant skin-to-skin contact; How do parent records compare to nurse records?	Sweden	Prospective Cohort	20 Parents	All ages	N/A	N/A	N/A	N/A	N/A	N/A	N/A	N/A
Bohnhorst	2004	Bradycardia and desaturation during skin-to-skin care: no relationship to hyperthermia	Germany	Pre-post	22 Newborns	<32 weeks, LBW: N/A cutoff	SSC	Once eligible: N/A definition	After measurement in incubator	2	1	N/A	N/A	N/A

Author	Year	Title	Country	Study design	Sample size	Newborns Characteristics	KMC components	Onset of SSC	Stability criteria for KMC initiation	Hours per day of KMC provision*	# of days of KMC provision*	Discharge criteria for Kangarooed baby	Follow-up duration	Follow-up location
Bohnhorst	2001	Skin-to-skin (kangaroo) care, respiratory control, and thermoregulation	Germany	Pre-post	22 Newborns	<32 weeks	SSC	Once eligible: N/A definition	After outcome measurement in incubator	2	1	N/A	N/A	N/A
Boju	2012	Short spell kangaroo mother care and its differential physiological influence in subgroups of preterm babies	India	Pre-post	86 Newborns	<37 weeks	N/A	N/A	N/A	1	1	N/A	N/A	N/A
Boo	2007	Short duration of skin-to-skin contact: Effects on growth and breastfeeding	Malaysia	RCT	126 Dyads	<1501g	SSC	Once eligible: N/A definition	Stable condition after adaptation to extra-uterine life	1	10	Clinically well, weighed ≥1750g, tolerate oral feed, weight gain at 10g/day, no apnea or bradycardia or desaturation for ≥5 consecutive days	N/A	N/A
Bosque	1995	Physiologic measures of kangaroo versus incubator care in a tertiary-level nursery	USA	Prospective Cohort	8 Dyads	All ages	SSC	Once eligible: N/A definition	N/A	4	21	N/A	N/A	N/A
Bramson	2010	Effect of early skin-to-skin mother--infant contact during the first 3 hours following birth on exclusive breastfeeding during the maternity hospital stay.	USA	Prospective Cohort	21842 Dyads	Full term	SSC, EBF	Immediately after birth	N/A	3	1	N/A	N/A	N/A

Author	Year	Title	Country	Study design	Sample size	Newborns Characteristics	KMC components	Onset of SSC	Stability criteria for KMC initiation	Hours per day of KMC provision*	# of days of KMC provision*	Discharge criteria for Kangarooed baby	Follow-up duration	Follow-up location
Brimdyr	2012	A Realistic Evaluation of Two Training Programs on Implementing Skin-to-Skin as a Standard of Care	Egypt	Focus group/ interview	40 Nurses and CHWs	N/A	SSC	Immediately after birth	N/A	1	1	N/A	N/A	N/A
Broughton	2013	The cost-savings of implementing kangaroo mother care in Nicaragua	Nicaragua	Chart review	98 Charts in a Facility	<37 weeks, <2500g	SSC, EBF, DC	N/A	N/A	N/A	N/A	N/A	N/A	N/A
Byaruhanga	2008	Perceptions among post-delivery mothers of skin-to-skin contact and newborn baby care in a periurban hospital in Uganda.	Uganda	Focus group/ interview	30 Mothers	All ages	SSC	Immediately after birth	N/A	N/A	N/A	N/A	N/A	N/A
Bystrova	2009	Early Contact versus Separation: Effects on Mother-Infant Interaction One Year Later	Russia	RCT	124 Dyads	Full term	SSC, EBF, FU	Immediately after birth	N/A	2	1	N/A	1,2,3,4,5,6, 9,12 mon. GA	Home
Bystrova	2007	The effect of Russian Maternity Home routines on breastfeeding and neonatal weight loss with special reference to swaddling	Russia	RCT	153 Dyads	All ages	SSC	Immediately after birth	N/A	N/A	N/A	N/A	N/A	N/A

Author	Year	Title	Country	Study design	Sample size	Newborns Characteristics	KMC components	Onset of SSC	Stability criteria for KMC initiation	Hours per day of KMC provision*	# of days of KMC provision*	Discharge criteria for Kangarooed baby	Follow-up duration	Follow-up location
Calais	2010	Skin-to-skin contact of fullterm infants: an explorative study of promoting and hindering factors in two Nordic childbirth settings	Sweden, Norway	Focus group/ interview	117 Mothers, 107 Fathers	Full term	SSC, DC, FU	Immediately after birth	N/A	N/A	N/A	Within 6-8 hours after birth	2 weeks after discharge	Home
Carbasse	2013	Safety and Effectiveness of Skin-to-Skin Contact in the NICU to Support Neurodevelopment in Vulnerable Preterm Infants	France	Pre-post	96 Newborns	<33 weeks	SSC	N/A	FiO2 <0.50, set ventilator respiratory rate <50 per min, no major respiratory event or requiring medical interventions in previous 12 hours	N/A	N/A	N/A	N/A	N/A
Carbonell	2010	Quality of maternal care: a comparison of preterm infants in Kangaroo mother care and full-term infants in regular care	Colombia	Intervention	93 Dyads	Premature: N/A cutoff, Full term	SSC, EBF, FU	Immediately after birth	No grave health state	N/A	N/A	LBW babies had to weigh 2400 g	3,7 mon.	Home
Carfoot	2005	A randomised controlled trial in the north of England examining the effects of skin-to-skin care on breast feeding	UK	RCT	204 Dyads	All ages	SSC, EBF, FU	Immediately after birth	N/A	N/A	N/A	N/A	2 week, 4 mon. after discharge	Home

Author	Year	Title	Country	Study design	Sample size	Newborns Characteristics	KMC components	Onset of SSC	Stability criteria for KMC initiation	Hours per day of KMC provision*	# of days of KMC provision*	Discharge criteria for Kangarooed baby	Follow-up duration	Follow-up location
Carfoot	2004	The value of a pilot study in breast-feeding research	UK	RCT	28 Mothers	≥36 weeks	SSC	Immediately after birth	N/A	N/A	N/A	N/A	N/A	N/A
Castiblanco López	2011	Vision of mothers in care of premature babies at home	Colombia	Focus group/ interview	8 Mothers	<36 weeks, 2320g	N/A	N/A	N/A	N/A	N/A	N/A	N/A	N/A
Castral	2008	The effects of skin-to-skin contact during acute pain in preterm newborns	Brazil	RCT	59 Newborns	30-<37 weeks	SSC	After 2min of baseline readings	N/A	0.283	1	N/A	N/A	N/A
Cattaneo	1998	Kangaroo mother care for low birth weight infants: a randomized controlled trial in different settings	Mexico, Indonesia, Ethiopia	RCT	7354 Newborns	1000-1999g	SSC,EBF,FU	Immediately after birth	N/A	20; mean	N/A	Indonesia; infants reaches 1500, weight gain, stable temperature for 3 days (N/A for other hospitals)	3,10,20, 30 d post discharge	Home
Charpak	1997	Kangaroo mother versus traditional care for newborn infants (less-than or equal to) 2000 grams: A randomized, controlled trial	Colombia	RCT	746 Newborns	<2000g	SSC, EBF,DC,FU	Once eligible: N/A definition	N/A	N/A	N/A	Regardless of weight or GA as soon as they medically stable and can suck swallow and breath	N/A	Home

Author	Year	Title	Country	Study design	Sample size	Newborns Characteristics	KMC components	Onset of SSC	Stability criteria for KMC initiation	Hours per day of KMC provision*	# of days of KMC provision*	Discharge criteria for Kangarooed baby	Follow-up duration	Follow-up location
Charpak	2001	A randomized, controlled trial of kangaroo mother care: results of follow-up at 1 year of corrected age	Colombia	RCT	777 Newborns	<2000g	SSC, DC,FU	Once eligible: N/A definition	Adapted to extra-uterine life, no infection or concomitant condition treated, sucking and swallowing properly, appropriate daily weight change	N/A	N/A	Neonatologist approval	term, 3, 6, 9, 12 mon. of CA	Home
Charpak	2006	Resistance to implementing Kangaroo Mother Care in developing countries, and proposed solutions	15 developing countries	Focus group/ interview	17 KMC coordinator, 15 Facilities	N/A	SSC, DC,FU	Immediately after birth	N/A	N/A	N/A	Regardless of weight or GA as soon as medically stable, can suck swallow and breath	Daily to twice weekly until regain birth weight, thriving at 15g /d	Health facility
Charpak	1994	Kangaroo Mother Program: an alternative way of caring for low birth weight infants? One year mortality in a two cohort study	Colombia	Prospective Cohort	332 Newborns	≤2000g	SSC, DC,FU	After surviving the neonatal period (2 mon. of life) and eligible for minimal care	Adapted to extra uterine life and any infection or associated condition has been properly treated	N/A	28.5	Adapted to extra-uterine life, no infection or associated condition, regardless of weight or GA	1,3,6,9,12 mon.th after discharge	Health facility
Chermont	2009	Skin-to-Skin Contact and/or Oral 25% Dextrose for Procedural Pain Relief for Term Newborn Infants	Brazil	RCT	640 Newborns	Full term	SSC	2 min before injection	N/A	0.083 (2 min) before injection	1	N/A	N/A	N/A
Chia	2006	The attitudes and practices of neonatal nurses in the use of kangaroo care	Australia	Focus group/ interview	34 Nurses	N/A	SSC	N/A	N/A	N/A	N/A	N/A	N/A	N/A

Author	Year	Title	Country	Study design	Sample size	Newborns Characteristics	KMC components	Onset of SSC	Stability criteria for KMC initiation	Hours per day of KMC provision*	# of days of KMC provision*	Discharge criteria for Kangarooed baby	Follow-up duration	Follow-up location
Chidambaram	2013	Effect of Kangaroo mother care in reducing pain due to heel prick among preterm neonates: a crossover trial	India	Crossover	50 Newborns	32-36 weeks, <2500g	SSC	N/A	N/A	≥0.25	1	N/A	N/A	N/A
Chiu	2009	Effect of early skin-to-skin contact on mother-preterm infant interaction through 18 months: Randomized controlled trial	USA	RCT	100 Dyads	Premature: N/A cutoff	SSC	N/A	N/A	N/A	N/A	N/A	At 6, 12, and 18 mon. GA	Health facility
Christensson	1998	Randomized study of skin-to-skin versus incubator care for rewarming low-risk hypothermic neonates	Zambia	RCT	80 Newborns	≥1500g	SSC	N/A	N/A	N/A	N/A	N/A	N/A	N/A
Chwo	2002	A randomized controlled trial of early kangaroo care for preterm infants: effects on temperature, weight, behavior, and acuity	Taiwan	RCT	34 Dyads	32-36 weeks	SSC	N/A	N/A	1	6	N/A	N/A	N/A

Author	Year	Title	Country	Study design	Sample size	Newborns Characteristics	KMC components	Onset of SSC	Stability criteria for KMC initiation	Hours per day of KMC provision*	# of days of KMC provision*	Discharge criteria for Kangarooed baby	Follow-up duration	Follow-up location
Colameo	2006	Kangaroo Mother Care in public hospitals in the State of Sao Paulo, Brazil: an analysis of the implementation process	Brazil	Cross sectional	28 Facilities	LBW: N/A cutoff	N/A	Once eligible: N/A definition	N/A	N/A	N/A	Clinical stability and weight of 2000g	Once in two or three days until reach 2500g	Health facility
Collados-Gomez	2011	Assessing the impact of kangaroo care on preterm infant stress	Spain	Chart review, pre-post	51 Newborns	29-34 weeks PMA	N/A	N/A	N/A	≥0.5	1	N/A	N/A	N/A
Cong	2012	Effects of skin-to-skin contact on autonomic pain responses in preterm infants	USA	Randomized crossover	26 Dyads	28-<33 weeks	SSC	N/A	N/A	0.25-0.5	N/A	N/A	N/A	N/A
Cong	2009	Kangaroo Care modifies preterm infant heart rate variability in response to heel stick pain: pilot study	USA	Randomized crossover	14 Dyads	30-32 weeks	SSC	After a NG tube or completed bottle feeding	N/A	1.5	1	N/A	N/A	N/A
Cong	2011	Randomized crossover trial of kangaroo care to reduce biobehavioral pain responses in preterm infants: a pilot study	USA	Randomized crossover	28 Newborns	30-32 weeks, 2-9 days GA	SSC	80 minutes before heel sticking procedure	N/A	1	1	N/A	N/A	N/A
Constantinou	1999	Effects of skin-to-skin holding on general movements of preterm	USA	Pre-post	10 Newborns	33-36 weeks, <2000g	SSC	After the first feeding of the day shift	N/A	N/A	1	N/A	N/A	N/A

Author	Year	Title	Country	Study design	Sample size	Newborns Characteristics	KMC components	Onset of SSC	Stability criteria for KMC initiation	Hours per day of KMC provision*	# of days of KMC provision*	Discharge criteria for Kangarooed baby	Follow-up duration	Follow-up location
		infants												
Crenshaw	2012	Use of a video-ethnographic intervention (PRECESS Immersion Method) to improve skin-to-skin care and breastfeeding rates	USA	Descriptive	261 Dyads, N/A for HCP	Full term	SSC	≤2min after birth	Healthcare provider determined if eligible for SSC after birth	N/A	1	N/A	N/A	N/A
Cruvinel	2007	Interaction between mothers and pre-term infants and mood changes: a comparison between the Kangaroo Mother Method and those observed on a visit to a Neonatal Intensive Care Unit	Brazil	Prospective Cohort	60 Mothers	<37 weeks, LBW: N/A cutoff	SSC,EBF	Once eligible: N/A definition	N/A	N/A	N/A	N/A	N/A	N/A
Dageville	2008	Very early neonatal apparent life-threatening events and sudden unexpected deaths: Incidence and risk factors	France	Case control	62968 Newborns	>36 weeks	SSC	N/A	N/A	N/A	N/A	N/A	N/A	N/A
Dala Sierra	1994	Experience Madre Kangaroo; Kangaroo mother experi-	Honduras	RCT	20 Newborns	<2000g	N/A	Immediately after birth	N/A	N/A	N/A	N/A	N/A	N/A

Author	Year	Title	Country	Study design	Sample size	Newborns Characteristics	KMC components	Onset of SSC	Stability criteria for KMC initiation	Hours per day of KMC provision*	# of days of KMC provision*	Discharge criteria for Kangarooed baby	Follow-up duration	Follow-up location
		ence												
Dalbye	2011	Mothers' experiences of skin-to-skin care of healthy full-term newborns - A phenomenology study	Sweden, Norway	Focus group/ interview	20 Mothers	Full term	SSC	Immediately after birth	N/A	N/A	N/A	N/A	N/A	N/A
Darmstadt	2006	Introduction of community-based skin-to-skin care in rural Uttar Pradesh, India	India	Intervention	1930 Newborns, 2063 Mothers	All ages	SSC	N/A	N/A	N/A	N/A	N/A	N/A	N/A
De Alencar	2009	Effect of kangaroo mother care on postpartum depression	Brazil	Crossover	180 Newborns	<2000g	SSC,EBF	When discharged from NICU	N/A	1-2	N/A	Weight gain of 10-15g/kg/d for 3 days	N/A	N/A
De Leeuw	1991	Physiological effects of kangaroo care in very small preterm infants	Netherlands	Pre-post	8 Newborns	27-29 weeks, 770-1465g	SSC	1 hour in the morning/ evening after eligibility	Could clinically be removed from incubator without risk	1	N/A	N/A	N/A	N/A
De Macedo	2007	The mood variation in mothers of preterm infants in Kangaroo mother care and conventional incubator care	Brazil	Intervention	90 Mothers	LBW: N/A cutoff, Full term	SSC	N/A	N/A	N/A	N/A	N/A	N/A	N/A
De Vonderweid	2003	Neonatal developmental care in Italian Neonatal Intensive Care Units	Italy	Pop based surveillance	109 Facilities	N/A	N/A	N/A	N/A	N/A	N/A	N/A	N/A	N/A

Author	Year	Title	Country	Study design	Sample size	Newborns Characteristics	KMC components	Onset of SSC	Stability criteria for KMC initiation	Hours per day of KMC provision*	# of days of KMC provision*	Discharge criteria for Kangarooed baby	Follow-up duration	Follow-up location
Diniz	2013	Effect of the kangaroo position on the electromyographic activity of preterm children: A follow-up study	Brazil	Intervention	30 Newborns	27-34 weeks	SSC	Immediately after the first electromyography reading	RR 30-60 /min, HR 120-160 bpm, oxygen saturation >0.89, no respiratory distress, no cyanosis or pallor or pain, tolerate food, un -assisted breathe weight >1250g	8-12	4	N/A	N/A	N/A
Duarte	2001	Kangaroo mother care: experience report	Brazil	Focus group/ interview	1 Mother	Premature: N/A cutoff	SSC	N/A	N/A	N/A	39	N/A	N/A	N/A
Eichel	2001	Kangaroo care: Expanding our practice to critically iII neonates	USA	Facility evaluation	1 Facility	N/A	N/A	N/A	N/A	N/A	N/A	N/A	N/A	N/A
Eka Pratiwi	2009	Effect of kangaroo method on the risk of hypothermia and duration of birth weight regain in low brith weight infants: A randomized control trial	Indonesia	RCT	98 Newborns	1500-2250g	N/A	Immediately after birth	N/A	N/A	N/A	N/A	N/A	N/A
Eleutério	2008	The imaginary of mothers about experiencing the mother-kangaroo method	Brazil	Focus group/ interview	9 Mothers	Premature: N/A cutoff	N/A	N/A	N/A	N/A	N/A	N/A	N/A	N/A
Engler	2002	Kangaroo care: national survey of practice, knowledge, barriers, and	USA	Facility evaluation	537 Facilities	N/A	N/A	N/A	N/A	N/A	N/A	N/A	N/A	N/A

Author	Year	Title	Country	Study design	Sample size	Newborns Characteristics	KMC components	Onset of SSC	Stability criteria for KMC initiation	Hours per day of KMC provision*	# of days of KMC provision*	Discharge criteria for Kangarooed baby	Follow-up duration	Follow-up location
		perceptions												
Entringer	2013	Budget impact of using the Kangaroo Method in neonatal care	Brazil	Chart review	1000 Newborns	>1250g	N/A	N/A	N/A	N/A	N/A	N/A	N/A	N/A
Erlandsson	2007	Skin-to-skin care with the father after cesarean birth and its effect on newborn crying and prefeeding behavior	Sweden	RCT	29 Father infant dyads	37-41 weeks, C-Section	SSC	Immediately after birth	N/A	N/A	1	N/A	N/A	N/A
Feldman	2002	Comparison of skin-to-skin (kangaroo) and traditional care: parenting outcomes and preterm infant development	Israel	Prospective cohort	146 Dyads	<1720g, <34 weeks	SSC	Once eligible: N/A definition	When medical situation stabilized and no longer needed ventilation	≥1	14	N/A	3 mon. CA at home, and 6 mon. CA at hospital	Home
Feldman	2002	Skin-to-Skin contact (Kangaroo care) promotes self-regulation in premature infants: sleep-wake cyclicity, arousal modulation, and sustained exploration	Israel	Prospective Cohort	146 Newborns	<1720g, <34 weeks	SSC	Once eligible: N/A definition	When medical situation stabilized, no longer needed ventilation	≥1	14	N/A	3 mon. of CA at home, 6 mon. CA at hospital	Home and health facility

Author	Year	Title	Country	Study design	Sample size	Newborns Characteristics	KMC components	Onset of SSC	Stability criteria for KMC initiation	Hours per day of KMC provision*	# of days of KMC provision*	Discharge criteria for Kangarooed baby	Follow-up duration	Follow-up location
Feldman	2003	Testing a family intervention hypothesis: the contribution of mother-infant skin-to-skin contact (kangaroo care) to family interaction, proximity, and touch	Israel	Prospective Cohort	146 Dyads	<1720g, <34 weeks	SSC	Once eligible: N/A definition	When their medical situation stabilized and they no longer needed ventilation	≥1	14	N/A	3 mon. of CA at home, 6 mon.CA at hospital	Home and health facility
Feldman	2014	Maternal-preterm skin-to-skin contact enhances child physiologic organization and cognitive control across the first 10 years of life	Israel	Prospective Cohort	146 Dyads	<1720g	SSC	Once eligible: N/A definition	N/A	1	14	N/A	N/A	N/A
Feldman	2003	Skin-to-skin contact (Kangaroo Care) accelerates autonomic and neurobehavioural maturation in preterm infants	Israel	Intervention	70 Newborns	25-33 weeks, <1650g	SSC	Once eligible: N/A definition	Stabilized medical situation	1	14	N/A	N/A	N/A
Ferber	2004	The effect of skin-to-skin contact (kangaroo care) shortly after birth on the neurobehavioral responses of the term newborn: a randomized, controlled	Israel	RCT	47 Dyads	Full term	SSC	N/A	N/A	N/A	N/A	N/A	N/A	N/A

Author	Year	Title	Country	Study design	Sample size	Newborns Characteristics	KMC components	Onset of SSC	Stability criteria for KMC initiation	Hours per day of KMC provision*	# of days of KMC provision*	Discharge criteria for Kangarooed baby	Follow-up duration	Follow-up location
		trial												
Ferber	2008	Neurobehavioural assessment of skin-to-skin effects on reaction to pain in preterm infants: a randomized, controlled within-subject trial	Israel	RCT	30 Newborns	28-34 weeks	SSC	10 min before blood test stick	N/A	N/A	N/A	N/A	N/A	N/A
Ferrarello	2014	Barriers to skin-to-skin care during the postpartum stay	USA	Focus group/ interview	15 Mothers, 14 Nurses	N/A	SSC	N/A	N/A	N/A	N/A	N/A	N/A	N/A
Fischer	1998	Cardiorespiratory stability of premature boys and girls during kangaroo care	USA	Pre-post	20 Newborns	25-32 weeks, <1610g	SSC	N/A	N/A	2	1	N/A	N/A	N/A
Flacking	2011	Positive effect of kangaroo mother care on long-term breastfeeding in very preterm infants	Sweden	Prospective Cohort	300 Newborns	<37 weeks	SSC	N/A	N/A	N/A	N/A	N/A	1,2,5,6 mon. of infant CA	Home

Author	Year	Title	Country	Study design	Sample size	Newborns Characteristics	KMC components	Onset of SSC	Stability criteria for KMC initiation	Hours per day of KMC provision[*]	# of days of KMC provision[*]	Discharge criteria for Kangarooed baby	Follow-up duration	Follow-up location
Flacking	2013	Influence of NICU co-care facilities and skin-to-skin contact on maternal stress in mothers of preterm infants	Sweden	Prospective Cohort	300 Dyads	<37 weeks	SSC	As soon as infant stability	N/A	N/A	N/A	N/A	N/A	N/A
Flynn	2010	Neonatal nurses' knowledge and beliefs regarding kangaroo care with preterm infants in an Irish neonatal unit	Ireland	Focus group/ interview	62 Nurses	N/A	N/A	N/A	N/A	N/A	N/A	N/A	N/A	N/A
Freire	2008	Evaluation of analgesic effect of skin-to-skin contact compared to oral glucose in preterm neonates	Brazil	RCT	105 Newborns	28-36 weeks	SSC	10 min before painful stimulus	N/A	≥0.2	1	N/A	N/A	N/A
Freitas	2007	Kangaroo Mother Method: newborn weight outcome	Brazil	Prospective cohort, descriptive	22 Newborns	N/A	N/A	N/A	N/A	N/A	N/A	N/A	N/A	N/A
Furlan	2003	Perception of parents in experiencing the kangaroo mother method	Brazil	Focus group/ interview	10 (5 couples)	Premature: N/A cutoff	SSC	Once eligible: N/A definition	N/A	10; mean	N/A	After achieve ≥2000g and breast-feeding is ok	Until 60 days of life	Health facility
Furman	2002	Correlates of lactation in mothers of very low birth weight infants	USA	Prospective cohort	149 Mothers	<33 weeks, 600-1499g	SSC	N/A	N/A	N/A	N/A	N/A	N/A	N/A

Author	Year	Title	Country	Study design	Sample size	Newborns Characteristics	KMC components	Onset of SSC	Stability criteria for KMC initiation	Hours per day of KMC provision*	# of days of KMC provision*	Discharge criteria for Kangarooed baby	Follow-up duration	Follow-up location
Gathwala	2010	Effect of Kangaroo Mother Care on physical growth, breastfeeding and its acceptability	India	RCT	110 Dyads	<1800g	SSC	N/A	Cardio-pulmon.ary stability	≥6	N/A	N/A	3 mon. GA	Home
Gathwala	2008	KMC facilitates mother baby attachment in low birth weight infants	India	RCT	110 Dyads	≤1800g	SSC	N/A	Cardio-pulmon.ary stability	≥6	N/A	N/A	3 mon. GA	Home
Ghavane	2012	Kangaroo Mother Care in Kangaroo ward for improving the growth and breastfeeding outcomes when reaching term gestational age in very low birth weight infants	India	RCT	149 Newborns	1500g	SSC,EBF,DC, FU	Once transferred to KMC ward	N/A	8	N/A	≥ 1300g or weight gain at ≥10g/day for 3 consecutive days	40 weeks GA	N/A
Gomez Papi	1998	[Kangaroo method in delivery room for full-term babies]	Spain	Prospective cohort, chart review	533 Dyads	Full term and delivered vaginally	SSC,EBF	Immediately after birth	N/A	≤2	1	N/A	N/A	N/A
Gontijo	2010	Evaluation of implementation of humanized care to low weight newborns: the Kangaroo Method	Brazil	Facility evaluation	293 Facilities	N/A	SSC,EBF	Once eligible: N/A definition	Stable clinical condition	N/A	N/A	N/A	N/A	N/A

Author	Year	Title	Country	Study design	Sample size	Newborns Characteristics	KMC components	Onset of SSC	Stability criteria for KMC initiation	Hours per day of KMC provision*	# of days of KMC provision*	Discharge criteria for Kangarooed baby	Follow-up duration	Follow-up location
Gontijo	2012	Evaluation of the implementation of Kangaroo Care by health administrators, professionals, and mothers of newborn infants	Brazil	Focus group/ interview	293 Facilities	N/A	N/A	N/A	N/A	N/A	N/A	N/A	N/A	N/A
Gonya	2013	Factors associated with maternal visitation and participation in skin-to-skin care in an all referral level IIIc NICU	USA	Focus group/ interview	32 Mothers	<27 weeks	SSC	N/A	N/A	N/A	N/A	N/A	N/A	N/A
Gray	2000	Skin-to-skin contact is analgesic in healthy newborns	USA	RCT	30 Newborns	Full term	SSC,EBF	10-15 min before heel stick	N/A	≤1	1	N/A	N/A	N/A
Gregson	2011	Kangaroo care in preterm or low birth weight babies in a postnatal ward	UK	Prospective Cohort	214 Newborns	All ages	SSC	N/A	N/A	N/A	N/A	N/A	6 weeks	Health facility
Gupta	2007	Kangaroo Mother Care (KMC) in LBW infants - A western Rajasthan experience	India	Facility evaluation	50 Newborns	<2000g	SSC	N/A	N/A	6-Apr	N/A	>1800g, gestation >34 weeks, mother' confidence in baby care, gaining weight, no illness, no IV medication, EBF	N/A	Home
Hake-Brooks	2008	Kangaroo care and breastfeeding of mother-preterm infant dyads 0-18 month:	USA	RCT	66 Dyads	32-36 weeks	SSC,EBF	N/A	N/A	N/A	5	N/A	N/A	N/A

Author	Year	Title	Country	Study design	Sample size	Newborns Characteristics	KMC components	Onset of SSC	Stability criteria for KMC initiation	Hours per day of KMC provision*	# of days of KMC provision*	Discharge criteria for Kangarooed baby	Follow-up duration	Follow-up location
		a randomized, controlled trial												
Haxton	2012	Implementing skin-to-skin contact at birth using the Iowa model: applying evidence to practice	USA	Intervention, qualitative	30 Mothers	All ages	SSC,EBF	Within one hour after birth	N/A	1	1	N/A	N/A	N/A
Heidarzadeh	2013	The Effect of Kangaroo Mother Care (KMC) on Breast Feeding at the Time of NICU Discharge	Iran	Prospective Cohort	251 Dyads	≥28 weeks	N/A	Immediately after birth	Stable situation (no more definition)	3-9	N/A	N/A	N/A	N/A
Heimann	2010	Impact of skin to skin care, prone and supine positioning on cardiorespiratory parameters and thermoregulation in premature infants	Germany	Crossover	18 Newborns	24-32 weeks, 750-2100g	SSC	After supine position, no more details	N/A	2	1	N/A	N/A	N/A
Heimann	2013	Infrared thermography for detailed registration of thermoregulation in premature infants	Germany	Pre-post	10 Newborns	<32 weeks, 720-1450g	SSC	N/A	N/A	1-1.5	1	N/A	N/A	N/A

Author	Year	Title	Country	Study design	Sample size	Newborns Characteristics	KMC components	Onset of SSC	Stability criteria for KMC initiation	Hours per day of KMC provision*	# of days of KMC provision*	Discharge criteria for Kangarooed baby	Follow-up duration	Follow-up location
Heinemann	2013	Factors affecting parents' presence with their extremely preterm infants in a neonatal intensive care room	Sweden	Focus group/ interview	7 Mothers, 6 Fathers	<27 weeks	SSC	N/A	N/A	N/A	N/A	N/A	N/A	N/A
Hendricks-Munoz	2010	Factors that influence neonatal nursing perceptions of family-centered care and developmental care practices	USA	Focus group/ interview	59 Nurses	N/A	SSC	N/A	N/A	N/A	N/A	N/A	N/A	N/A
Hendricks-Munoz	2013	Maternal and Neonatal Nurse Perceived Value of Kangaroo Mother Care and Maternal Care Partnership in the Neonatal Intensive Care Unit	USA	Qualitative	143 Mothers, 42 Nurses	<34 weeks	N/A	N/A	N/A	N/A	N/A	N/A	N/A	N/A
Hendricks-Munoz	2014	A Neonatal Nurse Training Program in Kangaroo Mother Care (KMC) Decreases Barriers to KMC Utilization in the NICU	USA	Prospective Cohort	30 Nurses	N/A	SSC	N/A	N/A	N/A	N/A	N/A	N/A	N/A

Author	Year	Title	Country	Study design	Sample size	Newborns Characteristics	KMC components	Onset of SSC	Stability criteria for KMC initiation	Hours per day of KMC provision*	# of days of KMC provision*	Discharge criteria for Kangarooed baby	Follow-up duration	Follow-up location
Hennig	2006	Health professional's knowledge and practices about	Brazil	Cross sectional	148 Doctors and nurses, 11 Facilities	LBW: N/A cutoff	N/A	Clinical stability	0.59 of the professionals did use (stable and not intubated) as criteria and 0.30 related allowed contact even when newborn stable but intubated.	N/A	N/A	N/A	N/A	N/A
Hernandez Molina	1988	Bio-psycho-social risk factors in children under one year of age of mother kangaroo program of the instituto materno infantil, Bogota, 1987	USA	RCT	8 Newborns	Premature: N/A cutoff	SSC	N/A	N/A	N/A	N/A	N/A	N/A	N/A
Hill	2010	Keeping newborns warm: beliefs, practices and potential for behaviour change in rural Ghana	Ghana	Focus group/ interview	635 Mothers, 14 Villages	All ages	SSC	N/A	N/A	N/A	N/A	N/A	N/A	N/A
Huang	2006	[Effect of very early kangaroo care on extra-uterine temperature adaptation in newborn infants with hypothermia problems]	Taiwan	RCT	164 Dyads	Full term	SSC	Immediately after birth	N/A	N/A	N/A	N/A	N/A	N/A

Author	Year	Title	Country	Study design	Sample size	Newborns Characteristics	KMC components	Onset of SSC	Stability criteria for KMC initiation	Hours per day of KMC provision*	# of days of KMC provision*	Discharge criteria for Kangarooed baby	Follow-up duration	Follow-up location
Ibe	2004	A comparison of kangaroo mother care and conventional incubator care for thermal regulation of infants < 2000 g in Nigeria using continuous ambulatory temperature monitoring	Nigeria	Crossover	13 Newborns, 11 Mothers and female relatives	1200-1999g	SSC	After enrollment	Able to tolerate handling without stress	12	N/A	N/A	N/A	N/A
Issah	2011	Maternal and neonatal survival and mortality in the Upper West Region of Ghana	Ghana	Prospective Cohort	695 Newborns, 5 Facilities	Premature: N/A cutoff, 588 LBW, 107 preterm	N/A	N/A	N/A	N/A	N/A	N/A	N/A	N/A
Jang	2008	The Effect of Kangaroo Care on Weight and Stress Hormone (Cortisol) in Premature Infants	South Korea	RCT	31 Newborns	<35 weeks, <1800g	N/A	N/A	N/A	3	3	N/A	N/A	N/A
Johnson	2007	Factors influencing implementation of kangaroo holding in a Special Care Nursery	USA	Focus group/ interview	17 Nurses	N/A	N/A	N/A	N/A	N/A	N/A	N/A	N/A	N/A
Johnson	2007	The Maternal Experience of Kangaroo Holding	Mid-Atlantic Region	Focus group/ interview	18 Mothers	N/A	N/A	N/A	N/A	≥1	N/A	N/A	N/A	N/A

Author	Year	Title	Country	Study design	Sample size	Newborns Characteristics	KMC components	Onset of SSC	Stability criteria for KMC initiation	Hours per day of KMC provision*	# of days of KMC provision*	Discharge criteria for Kangarooed baby	Follow-up duration	Follow-up location
Johnston	2012	Alternative female kangaroo care for procedural pain in preterm neonates: a pilot study	Canada	Randomized cross-over	41 Newborns	28-37 weeks	SSC	30 min before heel lance procedure	N/A	≤1	2	N/A	N/A	N/A
Johnston	2003	Kangaroo care is effective in diminishing pain response in preterm neonates	Canada	Randomized cross-over	74 Newborns, 3 Facilities	32-36 weeks	SSC	30 minutes before heel stick	Apgar> 6 at 5 min, un assisted breathing, no major congenital anomalies, no grade III or IV IVH, subsequent periventricular leukomalacia, not undergone surgery	0.7	1	N/A	N/A	N/A
Johnston	2008	Kangaroo mother care diminishes pain from heel lance in very preterm neonates: a crossover trial	Canada	Randomized cross-over	61 Newborns	28-31 weeks	SSC	≥15 min prior to heel lancing procedure	Apgar ≥ 6 at 5 minutes, un assisted breathing , no major congenital anomalies, no grade III or IV IVH or subsequent periventricular leukomalacia, not undergone surgery	0.5	N/A	N/A	N/A	N/A

Author	Year	Title	Country	Study design	Sample size	Newborns Characteristics	KMC components	Onset of SSC	Stability criteria for KMC initiation	Hours per day of KMC provision*	# of days of KMC provision*	Discharge criteria for Kangarooed baby	Follow-up duration	Follow-up location
Johnston	2011	Paternal vs maternal kangaroo care for procedural pain in preterm neonates: a randomized crossover trial	Canada	Randomized crossover	62 Newborns	28-36 weeks	SSC	At least 15 minutes before heel lance procedure	N/A	≤1	2	N/A	N/A	N/A
Kadam	2005	Feasibility of kangaroo mother care in Mumbai	India	RCT	89 Newborns	<1800g	SSC, EBF	N/A	Stable in cardiovascular status	≥1	N/A	weight-gain 3 consecutive days, normal unassisted temperature, well breastfed/spoon feeds, mother's confidence in baby care	N/A	N/A
Kaffashi	2013	An analysis of the kangaroo care intervention using neonatal EEG complexity: a preliminary study	USA	Prospective cohort	126 Newborns	Premature, Full term	SSC	N/A	N/A	N/A	56	N/A	N/A	N/A
Kambarami	1999	Kangaroo care for well low birth weight infants at Harare central hospital maternity unit - Zimbabwe	Zimbabwe	Facility evaluation	613 Dyads	Preterm: N/A cutoff, <2000g	N/A	N/A	Well preterm infants able to suck and weighed ≤2001	N/A	N/A	N/A	N/A	N/A

Author	Year	Title	Country	Study design	Sample size	Newborns Characteristics	KMC components	Onset of SSC	Stability criteria for KMC initiation	Hours per day of KMC provision*	# of days of KMC provision*	Discharge criteria for Kangarooed baby	Follow-up duration	Follow-up location
Kambarami	2002	Caregivers' perceptions and experiences of 'kangaroo care' in a developing country	Zimbabwe	Focus group/ interview	N/A Mothers	LBW:N/A cutoff,	N/A	N/A	The infants was nursed in incubators until there were stable and no longer required oxygen, then there were sent to KMC unit	N/A	N/A	Weighing 1000g	N/A	Health facility
Kambarami	1998	Kangaroo care versus incubator care in the management of well preterm infants-a pilot study	Zimbabwe	Intervention	74 Dyads	Premature: N/A cutoff, <1600g	SSC	After identifying a well preterm infants based on the neonatologist visit	Able to suck, no need to special care, oxygen or medication	N/A	N/A	N/A	N/A	N/A
Karlsson	2012	Early skin-to-skin care in extremely preterm infants: thermal balance and care environment	Sweden	Pre-post	27 Newborns	<27 weeks	SSC	Within a few days after birth when stable, after 30 min of monitoring in incubator	Neo-neonatologist deems respiratory, thermodynamically and thermal balance stable	≥1	N/A	N/A	N/A	N/A
Karlsson	1996	Skin to skin care: heat balance	Sweden	Prepost	9 Newborns	Full term	SSC	N/A	N/A	1	1	N/A	N/A	N/A
Kashaninia	2008	The effect of Kangaroo Care on behavioral responses to pain of an intramuscular injection in neonates	Iran	RCT	100 Newborns	Full term, Non LBW	SSC	10 minutes before procedure	N/A	N/A	N/A	N/A	N/A	N/A

Author	Year	Title	Country	Study design	Sample size	Newborns Characteristics	KMC components	Onset of SSC	Stability criteria for KMC initiation	Hours per day of KMC provision*	# of days of KMC provision*	Discharge criteria for Kangarooed baby	Follow-up duration	Follow-up location
Keshavarz	2010	Effects of kangaroo contact on some physiological parameters in term neonates and pain score in mothers with cesarean section	Iran	RCT	160 Dyads	Full term	SSC	2 hours after C/S	Healthy baby after delivery	3	N/A	N/A	N/A	N/A
Kim	1999	Physiological Effects of Kangaroo Care(skin-to-skin care) in Small Preterm Infants and Their Parents	N/A	Prospective Cohort	10 Newborns	<2000g	N/A	Immediately after birth	N/A	N/A	N/A	N/A	N/A	N/A
Koo	2000	The Effects of Kangaroo Care on Body Weight, Physiologic Responses and Behavioral States in Premature Infants	N/A	RCT	32 Newborns	28.5-33.5 weeks	N/A	N/A	N/A	8	25	N/A	N/A	N/A
Kostandy	2008	Kangaroo Care (skin contact) reduces crying response to pain in preterm neonates: pilot results	USA	Randomized crossover	10 Newborns	30-32 weeks	SSC	30 min before the baseline phase (heel stick)	No known congenital anomaly grade III or IV IVH; no history of surgery, drug exposure, any tissue breakdown or heel in-flammation	0.83	1	N/A	N/A	N/A
Kymre	2013	Balancing preterm infants' developmental needs with parents' readiness for skin-to-skin	Sweden, Norway, Denmark	Focus group/ interview	18 Nurses	N/A	SSC	N/A	N/A	N/A	N/A	N/A	N/A	N/A

Author	Year	Title	Country	Study design	Sample size	Newborns Characteristics	KMC components	Onset of SSC	Stability criteria for KMC initiation	Hours per day of KMC provision*	# of days of KMC provision*	Discharge criteria for Kangarooed baby	Follow-up duration	Follow-up location
		care: A phenomenological study												
Kyoko	2006	Implementation of Kangaroo Care Immediately After Childbirth	Japan	Survey at one year	106 Mothers	N/A	N/A	Immediately after birth	N/A	N/A	N/A	N/A	N/A	N/A
Lamy Filho	2008	Evaluation of the neonatal outcomes of the kangaroo mother method in Brazil	Brazil	Prospective Cohort	985 Newborns, 16 Facilities	500-1749g	SSC, EBF	N/A	Clinical stability	N/A	N/A	N/A	N/A	N/A
Lau	2007	Ethnic/racial diversity, maternal stress, lactation and very low birthweight infants	USA	Descriptive	163 Mothers	26-29 weeks	SSC	N/A	N/A	N/A	N/A	N/A	N/A	N/A
Lee	2011	The Effects of Kangaroo Care on Maternal Self-esteem and Premature Infants' Physiological Stability	South Korea	Pre-post	34 Dyads	>32 weeks,1000-2000g	SSC	After feeding	N/A	0.5	14	N/A	N/A	N/A
Lee	2012	Clinician perspectives on barriers to and opportunities for skin-to-skin contact for premature infants in neonatal intensive care units	USA	Focus group/ interview	69 HCPs, 11 Facilities	N/A	SSC	N/A	Determined by age, weight, respiratory distress, blood pressure, temperature, apnea, bradycardia, oxygen desaturation events	N/A	N/A	N/A	N/A	N/A

Author	Year	Title	Country	Study design	Sample size	Newborns Characteristics	KMC components	Onset of SSC	Stability criteria for KMC initiation	Hours per day of KMC provision*	# of days of KMC provision*	Discharge criteria for Kangarooed baby	Follow-up duration	Follow-up location
Lee	2007	Effects of Kangaroo Care on anxiety, maternal role confidence, and maternal infant attachment of mothers who delivered preterm infants	South Korea	Pre-post	43 Mothers	Premature: N/A cutoff,	N/A	N/A	N/A	N/A	4	N/A	N/A	N/A
Legault	1995	Comparison of kangaroo and traditional methods of removing preterm infants from incubators	Canada	RCT, pre-post, cross over	61 Dyads	Premature: N/A cutoff, 1000-1800g	SSC	Once eligible: N/A definition	≤1 respiratory pause or one episode of bradycardia in the past 5 days, started weight gain	0.5	1	N/A	N/A	N/A
Lemmen	2013	Kangaroo care in a neonatal context: parents' experiences of information and communication of nurse-parents	Sweden	Focus group/ interview	15 Newborns, 12 Families	24-35 weeks	SSC	N/A	N/A	N/A	N/A	N/A	N/A	N/A
Leonard	2008	Parents' Lived Experience of Providing Kangaroo Care to their Preterm Infants	South Africa	Focus group/ interview	6 Parents	Premature: N/A cutoff	N/A	N/A	N/A	N/A	N/A	N/A	N/A	N/A
Lerthamtewe	2000	The effects of kangaroo care on body temperature, body weight and maternal satisfaction in premature	N/A	RCT	30 Newborns	33-35 weeks PCA	N/A	N/A	N/A	1.5-2	N/A	N/A	N/A	N/A

Author	Year	Title	Country	Study design	Sample size	Newborns Characteristics	KMC components	Onset of SSC	Stability criteria for KMC initiation	Hours per day of KMC provision*	# of days of KMC provision*	Discharge criteria for Kangarooed baby	Follow-up duration	Follow-up location
		infants												
Lima	2000	Feasibility, acceptability and cost of kangaroo mother care in Recife, Brazil	Brazil	Facility evaluation	114 Newborns	1000-1750g	SSC, EBF, FU	After stabilization	Unassisted breathing	N/A	N/A	N/A	N/A	Home and health facility
Lincetto	1998	Impact of season and discharge weight on complications and growth of Kangaroo Mother Care treated low birth weight infants in Mozambique	Mozambique	Prospective Cohort	246 Newborns	<2000g	SSC, EBF,DC, FU	Stabilized health condition, sucking reflex, thermoregulation, mother's condition enabling her to care for the LBW infant, cessation of infant IV, oxygen, and phototherapy, no feeding by NG tube	Same criteria as onset of SSC	>20	N/A	Weight ≥1500g, weight gain through 3 consecutive days	Twice weekly for 1500-1700g, once weekly for 1700-2000g, and fortnightly for over 2000g babies	Health facility

Author	Year	Title	Country	Study design	Sample size	Newborns Characteristics	KMC components	Onset of SSC	Stability criteria for KMC initiation	Hours per day of KMC provision*	# of days of KMC provision*	Discharge criteria for Kangarooed baby	Follow-up duration	Follow-up location
Lincetto	2000	Kangaroo mother care with limited resources	Mozambique	Facility evaluation	32 Newborns	≤1800g	SSC, EBF,DC, FU	As soon as possible after admission to all infants<1800 g, irrespective of GA, place of birth, and health condition	N/A	N/A	N/A	Weight ≥1500g, weight gain for ≥ 3 consecutive days	Twice a week until infant reached 2500 g	Health facility
Lizarazo-Medina	2012	The kangaroo mothers' program: a simple and cost-effective alternative for protecting the premature newborn or low-birth-weight babies	Colombia	Retrospective cohort	374 Newborns	Premature and LBW:N/A cutoff	SSC	N/A	N/A	N/A	240	N/A	N/A	N/A
Ludington-Hoe	2000	Kangaroo care compared to incubators in maintaining body warmth in preterm infants	USA	RCT, pre/post	29 Newborns	26-35 weeks, 770-2710g	SSC, EBF	The KC period began once the infant had been transferred from the incubator to the mother who was sitting in an upright chair beside the	N/A	2.5-3.83	1	N/A	N/A	N/A

Author	Year	Title	Country	Study design	Sample size	Newborns Characteristics	KMC components	Onset of SSC	Stability criteria for KMC initiation	Hours per day of KMC provision*	# of days of KMC provision*	Discharge criteria for Kangarooed baby	Follow-up duration	Follow-up location
								incubator						
Ludington-Hoe	2001	Kangaroo mother care during phototherapy: effect on bilirubin profile	USA	RCT	30 Newborns	30-35 weeks, <2500g	SSC	Once eligible: N/A definition	N/A	1	10	N/A	N/A	N/A
Ludington-Hoe	1999	Birth-related fatigue in 34-36-week preterm neonates: rapid recovery with very early kangaroo (skin-to-skin) care	Colombia	Descriptive	6 Newborns	34-36 weeks	SSC, EBF	Immediately after birth	N/A	6	1	N/A	N/A	N/A
Ludington-Hoe	2006	Neurophysiologic assessment of neonatal sleep organization: Preliminary results of a randomized, controlled trial of skin contact with preterm infants	USA	RCT	28 Newborns	28-32 weeks	SSC	Once eligible: N/A definition	Apgar >6 at 5 min	2-3	1	N/A	N/A	N/A
Ludington-Hoe	2004	Randomized controlled trial of kangaroo care: cardiorespiratory and thermal	N/A	RCT, pre/post	24 Newborns	32-36 weeks	SSC, EBF	After pretest phase, determined by breast	No medication in the last 24 hours	3	1	N/A	N/A	N/A

Author	Year	Title	Country	Study design	Sample size	Newborns Characteristics	KMC components	Onset of SSC	Stability criteria for KMC initiation	Hours per day of KMC provision*	# of days of KMC provision*	Discharge criteria for Kangarooed baby	Follow-up duration	Follow-up location
		effects on healthy preterm infants						feeding						
Ludington-Hoe	2005	Skin-to-skin contact (Kangaroo Care) analgesia for preterm infant heel stick	El Salvador	Randomized cross-over	23 Newborns	<37 weeks	SSC	3 hours before heel stick	N/A	3	1	N/A	N/A	N/A
Lyngstad	2014	Does skin-to-skin contact reduce stress during diaper change in preterm infants?	Norway	Randomized cross-over	19 Newborns	28-34 weeks	SSC	N/A	N/A	N/A	2	N/A	N/A	N/A
Maastrup	2010	Extremely preterm infants tolerate skin-to-skin contact during the first weeks of life	Sweden, Norway, Denmark	Pre-post	22 Newborns	<28 weeks	SSC	After pre-test	N/A	N/A	1	N/A	N/A	N/A
Maastrup	2012	Breastfeeding Support in Neonatal Intensive Care: A National Survey	Denmark	Facility evaluation	19 Facilities	N/A	SSC	18 out of 19 aimed within 24 h postpartum for stable preterm infant	N/A	N/A	N/A	N/A	N/A	N/A
Mahmood	2011	Effect of mother-infant early skin-to-skin contact on breastfeeding status: a randomized controlled trial	Pakistan	RCT	183 Dyads	≥37 weeks, ≥2500g	SSC, EBF	Once eligible: N/A definition	No respiratory stress and weight >2500 g	≥0.75-2	30	N/A	N/A	N/A

Author	Year	Title	Country	Study design	Sample size	Newborns Characteristics	KMC components	Onset of SSC	Stability criteria for KMC initiation	Hours per day of KMC provision*	# of days of KMC provision*	Discharge criteria for Kangarooed baby	Follow-up duration	Follow-up location
Mallet	2007	[Skin to skin contact in neonatal care: knowledge and expectations of health professionals in 2 neonatal intensive care units]	France	Focus group/ interview	21 Doctors, 100 Paramedical staff	N/A	N/A	N/A	N/A	N/A	N/A	N/A	N/A	N/A
Marin Gabriel	2010	Randomized controlled trial of early skin-to-skin contact: effects on the mother and the newborn	Spain	RCT	274 Dyads	35-42 weeks	SSC, EBF, FU	Immediately after birth	No need to resuscitation or intubation, no meconium fluid	2	1	N/A	N/A	Home
Marin Gabriel	2008	Evaluation of pain in a neonatal intensive care unit during endocrine-metabolic tests	Spain	RCT	54 Newborns	Premature: N/A cutoff	SSC	N/A	N/A	≤1	1	N/A	N/A	N/A
Martins	2008	Living in the other side of kangaroo method: the mother's experience	Brazil	Focus group/ interview	5 Mothers	N/A	N/A	N/A	N/A	N/A	N/A	N/A	N/A	N/A
McMaster	2000	Kangaroo care in Port Moresby, Papua New Guinea	Papua New Guinea	Chart review, facility evaluation	109 Newborns	<1500g	SSC	N/A	N/A	N/A	N/A	Baby fully breast fed, gained weight, mother fully confident	N/A	Home
Messmer	1997	Effect of kangaroo care on sleep time for neonates	USA	Pre-post	20 Dyads	26-37 weeks, 750-1500g	SSC	After pre period	N/A	1	1	N/A	N/A	N/A

Author	Year	Title	Country	Study design	Sample size	Newborns Characteristics	KMC components	Onset of SSC	Stability criteria for KMC initiation	Hours per day of KMC provision*	# of days of KMC provision*	Discharge criteria for Kangarooed baby	Follow-up duration	Follow-up location
Miles	2006	A controlled trial of skin-to-skin contact in extremely preterm infants	UK	RCT	78 Newborns	<32 weeks	SSC	Once eligible: N/A definition	not ventilated, no repeated apnea or bradycardia, not on inotropic support, a stable core-peripheral temperature gap of less than 2 degrees C	0.33	28	N/A	4 mo and 12 mo post-birth and 12 mo from term	Health facility
Miltersteiner	2003	Physiological responses of the Kangaroo Mother Position in low birth weight, spontaneous ventilating premature babies	Brazil	Pre-post	46 Dyads	24-37 weeks, <2000g	SSC, EBF	Once eligible: N/A definition	One hour after feeding	1	Depends on mother's length of stay	N/A	N/A	N/A
Mitchell	2013	Does daily kangaroo care provide sustained pain and stress relief in preterm infants?	USA	RCT	54 Newborns	27-30 weeks, ≥1000g	SSC	5 days of life	N/A	≥2	5	N/A	N/A	N/A
Mitchell	2013	Effects of daily kangaroo care on cardio-respiratory parameters in preterm infants	USA	RCT	38 Newborns	27-30 weeks, 1000-1500g	SSC	5 day of life	N/A	2	5	N/A	N/A	N/A

Author	Year	Title	Country	Study design	Sample size	Newborns Characteristics	KMC components	Onset of SSC	Stability criteria for KMC initiation	Hours per day of KMC provision*	# of days of KMC provision*	Discharge criteria for Kangarooed baby	Follow-up duration	Follow-up location
Mohammed El-Nagger	2013	Effect of kangaroo mother care on premature infants' physiological, behavioral and psychosocial outcomes in Ain Shams maternity and Gynecological Hospital, Cairo, Egypt	Egypt	Pre-post	50 Dyads	Premature and LBW but N/A cutoff	SSC	Once eligible: N/A definition	N/A	1	1	N/A	N/A	N/A
Monasterolo	1998	Kangaroo care in preterm newborn infants admitted to a Neonatal Intensive Care Unit	Spain	Prospective Cohort	38 Newborns	Premature: N/A cutoff	SSC, EBF	N/A	N/A	1-8	N/A	N/A	N/A	N/A
Moon	2000	The Effects of Kangaroo Care on Anxiety and Confidence and Gratification of Mothering Role in Mothers of Low Birth Weight Infants	South Korea	RCT	30 Newborns	LBW:N/A cutoff	N/A	N/A	N/A	≥ 0.67	26	N/A	N/A	N/A
Moreira	2009	Kangaroo mother program and the relationship mother-baby: qualitative research in a public maternity of Betim city	Brazil	Focus group/ interview	8 Mothers	30-32 weeks, <2000g	SSC	Once eligible: N/A definition	Weight gain and stabilization of the baby	N/A	N/A	Mother prepared to baby care, weight ≥1500 g , suction capacity, EBF	N/A	Health facility

Author	Year	Title	Country	Study design	Sample size	Newborns Characteristics	KMC components	Onset of SSC	Stability criteria for KMC initiation	Hours per day of KMC provision*	# of days of KMC provision*	Discharge criteria for Kangarooed baby	Follow-up duration	Follow-up location
Morelius	2005	Salivary cortisol and mood and pain profiles during skin-to-skin care for an unselected group of mothers and infants in neonatal intensive care	Sweden	Pre-post	17 Dyads	33 weeks, 495-2590g	SSC	N/A	N/A	1	2	N/A	N/A	N/A
Morelius	2012	Time of initiation of skin-to-skin contact in extremely preterm infants in Sweden	Sweden	Pop based surveillance	520 Newborns	<27 weeks	SSC	N/A	N/A	N/A	N/A	N/A	N/A	N/A
Morgan	2011	Should Neonates Sleep Alone?	South Africa	Randomized cross-over	16 Dyads	Full term	SSC	N/A	N/A	1	1	N/A	N/A	N/A
Muddu	2013	Knowledge and awareness about benefits of kangaroo mother care	India	Pre-post, qualitative	46 Dyads	28-37 weeks	N/A	After asking questions from mothers as baseline knowledge	N/A	1	1	N/A	N/A	N/A
Nagai	2010	Earlier versus later continuous Kangaroo Mother Care (KMC) for stable low-birth-weight infants: a randomized controlled trial	Madagascar	RCT	73 Newborns	<2500g	SSC, EBF	Immediately after birth	N/A	N/A	N/A	N/A	N/A	N/A

Author	Year	Title	Country	Study design	Sample size	Newborns Characteristics	KMC components	Onset of SSC	Stability criteria for KMC initiation	Hours per day of KMC provision*	# of days of KMC provision*	Discharge criteria for Kangarooed baby	Follow-up duration	Follow-up location
Nagai	2011	Long-term effects of earlier initiated continuous Kangaroo Mother Care (KMC) for low-birth-weight (LBW) infants in Madagascar	Madagascar	RCT	73 Newborns	<2500g	SSC, EBF	As soon as possible within 24 h GA or after completely settled	Oxygen saturation, 0.95 or more, HR >100 bpm, RR <60 pm, capillary refilling time<3sec	N/A	N/A	N/A	N/A	N/A
Nanavati	2013	Effect of Kangaroo Mother Care Expressed Breast Milk Administration on Pain Associated with Removal of Adhesive Tapes in Very Low Birth Weight Neonates: A Randomized Controlled Trial	India	RCT	50 Newborns	LBW:N/A cutoff	SSC	15 min before removal of tape	N/A	0.25	N/A	N/A	N/A	N/A
Ndiaye	2006	[Efficiency of kangaroo care on thermoregulation and weight gain of a preterm newborn cohort in Dakar]	Senegal	Chart review	56 Newborns	Premature: N/A cutoff, <2000g	SSC, EBF, FU	N/A	N/A	N/A	N/A	N/A	N/A	N/A
Neu	2004	Kangaroo care: is it for everyone?	USA	Focus group/ interview	37 Mothers	31-34 weeks	N/A	N/A	N/A	N/A	N/A	N/A	N/A	N/A
Neu	2010	Maternal Holding of Preterm Infants During the Early Weeks After Birth and Dyad Interaction at Six months	USA	RCT	87 Dyads	32-34 weeks	SSC	Four weeks after birth of infant	N/A	≥1	N/A	N/A	Twice a week for 2 weeks followed by weekly visit for 6 weeks	Home and health facility

Author	Year	Title	Country	Study design	Sample size	Newborns Characteristics	KMC components	Onset of SSC	Stability criteria for KMC initiation	Hours per day of KMC provision*	# of days of KMC provision*	Discharge criteria for Kangarooed baby	Follow-up duration	Follow-up location
Neu	1999	Parents' perception of skin-to-skin care with their preterm infants requiring assisted ventilation	N/A	Focus group/ interview	8 Mothers, 1 Father	Premature: Mother, Father	SSC	N/A	N/A	1	2	N/A	N/A	N/A
Neu	2014	Effect of holding on co-regulation in preterm infants: A randomized controlled trial	USA	RCT	79 Newborns	32-35 weeks	N/A	N/A	N/A	1	3 at baseline & week 2 & week 9	N/A	Once a week for 8 weeks	Home
Neu	2013	Influence of holding practice on preterm infant development	USA	RCT	87 Dyads	32-35 weeks	N/A	N/A	N/A	N/A	N/A	N/A	N/A	N/A
Nguah	2011	Perception and practice of Kangaroo Mother Care after discharge from hospital in Kumasi, Ghana: a longitudinal study	Ghana	Prospective Cohort	195 Dyads	1000-2000g	SSC, EBF, FU	After admission in hospital and if mother was willing	no life threatening conditions or major congenital abnormalities or requiring ICU	N/A	N/A	N/A	1, 2, 3, and 4 weeks after discharge	Health facility
Niela-Vilen	2013	Early physical contact between a mother and her NICU-infant in two university hospitals in Finland	Finland	Prospective cohort, qualitative	170 Dyads, 381 Staff	All NICU newborns	N/A	Immediately after birth	N/A	N/A	N/A	N/A	N/A	N/A

Author	Year	Title	Country	Study design	Sample size	Newborns Characteristics	KMC components	Onset of SSC	Stability criteria for KMC initiation	Hours per day of KMC provision*	# of days of KMC provision*	Discharge criteria for Kangarooed baby	Follow-up duration	Follow-up location
Nimbalkar	2013	Kangaroo Mother Care in reducing pain in preterm neonates on heel prick	India	RCT	50 Newborns	32-36 weeks, <2500g	SSC	15 minutes before heel stick	Vitally stable, unassisted breathing or on CPAP, no neurological signs, not received analgesics or sedatives within last 24 h	0.5	1	N/A	N/A	N/A
Nimbalkar	2012	Reduced duration of cpap in preterm babies receiving kangaroo care within an hour of birth - Randomized trial	India	RCT	16 Newborns	26-32 weeks	N/A	N/A	N/A	N/A	N/A	N/A	N/A	N/A
Nimbalkar	2014	Effect of early skin-to-skin contact following normal delivery on incidence of hypothermia in neonates more than 1800g: a randomized control trial	India	RCT	100 Newborns	Premature: N/A cutoff, ≥1800g	SSC, EBF	After immediate post delivery care was done, at about 30min-1hr after birth	no need for resuscitation measured at birth	≥1	1	N/A	N/A	N/A

Author	Year	Title	Country	Study design	Sample size	Newborns Characteristics	KMC components	Onset of SSC	Stability criteria for KMC initiation	Hours per day of KMC provision*	# of days of KMC provision*	Discharge criteria for Kangarooed baby	Follow-up duration	Follow-up location
Nirmala	2006	Kangaroo Mother Care: Effect and perception of mothers and health personnel	India	Qualitative	50 Dyads	Premature: N/A cutoff, 1070-2460g	N/A	N/A	N/A	≥0.5	N/A	N/A	N/A	N/A
Nyqvist	2008	Application of the baby friendly hospital initiative to neonatal care: suggestions by Swedish mothers of very preterm infants	Sweden	Focus group/ interview	13 Mothers	<32 weeks	SSC, DC, FU	N/A	N/A	N/A	N/A	no apnea, unassisted normal temperature, partial oral feeding, adequate weight gain, no illness, parental' confidence in caring infants, ability to perform tube and/or cup feeding	N/A	Home at initial, then clinic
Ohgi	2002	Comparison of kangaroo care and standard care: behavioral organization, development, and temperament in healthy, low-birth-weight infants through 1 year	Japan	Intervention	53 Newborns	1501-2099g	SSC, EBF, FU	As soon as they could tolerate handling outside incubator	As soon as they could tolerate handling outside incubator	N/A	N/A	N/A	6 and 12 mon. CA	Home

Author	Year	Title	Country	Study design	Sample size	Newborns Characteristics	KMC components	Onset of SSC	Stability criteria for KMC initiation	Hours per day of KMC provision*	# of days of KMC provision*	Discharge criteria for Kangarooed baby	Follow-up duration	Follow-up location
Okan	2010	Analgesic effects of skin-to-skin contact and breastfeeding in procedural pain in healthy term neonates	Turkey	RCT	108 Newborns	Full term	SSC	15 minutes before heel stick	N/A	≤1	1	N/A	N/A	N/A
Ortiz	2006	Attachment Relationship Between Adolescent Mothers and Their 'Kangaroo' Babies'	Colombia	Prospective Cohort	40 Dyads	All ages	N/A	N/A	N/A	N/A	N/A	N/A	N/A	N/A
Ortiz	2006	Attachment relationships between adolescent mothers and their 'kangaroo' babies	Colombia	Retrospective cohort	40 Dyads	Premature: N/A cutoff, <2200g in KMC group, Full term in Control group	N/A	N/A	N/A	N/A	N/A	N/A	N/A	Health facility

Author	Year	Title	Country	Study design	Sample size	Newborns Characteristics	KMC components	Onset of SSC	Stability criteria for KMC initiation	Hours per day of KMC provision*	# of days of KMC provision*	Discharge criteria for Kangarooed baby	Follow-up duration	Follow-up location
Park	2014	Practical application of kangaroo mother care in preterm infants: clinical characteristics and safety of kangaroo mother care	South Korea	Intervention	31 Newborns	25-32 weeks	SSC, EBF	No apnea, none or self limiting (3 apnea = 1 h), bradycardia, none or > 85–100 beats/min with self-recovery, desaturation, none or = 0.85 with self-recovery and/or FiO2 = 0.4], clinically acceptable blood pressure and others (no arterial line, no chest tube, no intensive phototherapy and no sudden deterioration in condition within the past 2 days	The same as SSC onset criteria	0.5-1.5	1–2 times per week until the transition	N/A	N/A	N/A

Author	Year	Title	Country	Study design	Sample size	Newborns Characteristics	KMC components	Onset of SSC	Stability criteria for KMC initiation	Hours per day of KMC provision*	# of days of KMC provision*	Discharge criteria for Kangarooed baby	Follow-up duration	Follow-up location
Parmar	2009	Experience with Kangaroo mother care in a neonatal intensive care unit (NICU) in Chandigarh, India	India	Retrospective cohort	135 Newborns	26-37 weeks, 550-2500g	SSC	N/A	Out of critical illness	N/A	N/A	N/A	N/A	N/A
Pattinson	2006	Does kangaroo mother care save lives?	South Africa	Chart review	78 Facilities	1000-1999g	N/A	N/A	N/A	N/A	N/A	N/A	N/A	N/A
Pattinson	2005	Implementation of kangaroo mother care: a randomized trial of two outreach strategies	South Africa	RCT	34 Facilities	N/A	N/A	N/A	N/A	N/A	N/A	N/A	N/A	N/A
Pejovic	2013	Unexpected collapse of healthy newborn infants: Risk factors, supervision and hypothermia treatment	Sweden	Case control, chart review	26 Newborns	≥35 weeks	SSC	N/A	N/A	N/A	N/A	N/A	N/A	N/A
Penalva	2006	Descriptive study of the clinical and nutritional profile and follow-up of premature babies in a Kangaroo Mother Care Program	Brazil	Descriptive, chart review	70 Newborns	mean 32.5 weeks, mean 1560g	SSC, EBF, FU	Around 14 days	N/A	24	11.5; mean	N/A	12 mon.	Health facility
Phillips	2012	Prevention of postpartum smoking relapse in mothers of infants in the neonatal intensive care unit	USA	RCT	54 Mothers	All NICU newborns	SSC	N/A	N/A	N/A	N/A	N/A	N/A	N/A

Author	Year	Title	Country	Study design	Sample size	Newborns Characteristics	KMC components	Onset of SSC	Stability criteria for KMC initiation	Hours per day of KMC provision*	# of days of KMC provision*	Discharge criteria for Kangarooed baby	Follow-up duration	Follow-up location
Priya	2004	Kangaroo care for low birth weight babies	India	Crossover	30 Dyads	LBW:N/A cutoff	SSC	After the routine care was observed and data were collected	N/A	2	2	N/A	N/A	N/A
Quasem	2003	Adaptation of kangaroo mother care for community-based application	Bangladesh	Focus group/ interview	35 Mothers	All ages	SSC	N/A	N/A	N/A	N/A	N/A	N/A	N/A
Rajai	2012	Comparison of effect of kangaroo care and mother holding on sleep and waking states of preterm neonates	Iran	RCT, Pre-post	70 Newborns	32-37 weeks	SSC	After pre-intervention period	N/A	1.16	N/A	N/A	N/A	N/A
Ramanathan	2001	Kangaroo Mother Care in very low birth weight infants	India	RCT	28 Newborns	<1500g	N/A	Once eligible: N/A definition	Cardio-pumon.ary and temperature stability	≥4	N/A	1401 g, GA ≥34 weeks, mothers confidence to go home, weight gain, no medication or illness, and EBF	N/A	N/A
Reynolds	2013	Parental presence and holding in the neonatal intensive care unit and associations with early neurobehavior	USA	Prospective Cohort	81 Newborns	≤30 weeks	SSC	N/A	N/A	N/A	N/A	N/A	N/A	N/A
Roberts	2000	A comparison of kangaroo mother care and conventional cuddling care	Australia	RCT	30 Dyads	≥30 weeks, SGA	SSC	Once eligible: N/A definition	N/A	2	5	N/A	N/A	N/A

Author	Year	Title	Country	Study design	Sample size	Newborns Characteristics	KMC components	Onset of SSC	Stability criteria for KMC initiation	Hours per day of KMC provision*	# of days of KMC provision*	Discharge criteria for Kangarooed baby	Follow-up duration	Follow-up location
Rodrigues	2009	Trial gain of weight and hospital length stay of the low birth weight preterm infant in assistance for kangaroo mother care	Brazil	Chart review	120 Newborns	<37 weeks, <2000g	SSC	Once eligible: N/A definition	N/A	2	Depends on mother's length of stay	Achieve more than 2000g	N/A	N/A
Rodriguez	2007	Good short-term outcomes of kangaroo mother care in infants with a low birth weight in a rural South African hospital	South Africa	Descriptive	87 Dyads	1000-2500g	N/A	Once eligible: N/A definition	Without oxygen more of day, hemodynamically stable, no IV fluids	N/A	N/A	Mother comfortable, infant gaining weight (154 g/week).	N/A	N/A
Rojas	2003	Somatic growth of preterm infants during skin-to-skin care versus traditional holding: a randomized, controlled trial	USA	RCT	60 Newborns	≤32 weeks, ≤1500g	SSC	Once eligible: N/A definition	Hemodynamically stable	8	4	N/A	N/A	N/A
Roller	2005	Getting to know you: mothers' experiences of kangaroo care	USA	Focus group/ interview	10 Mothers	32-37 weeks	SSC	N/A	N/A	N/A	N/A	N/A	N/A	N/A
Sá	2010	Interpersonal relationships between professionals and mothers of premature from Kangaroo-Unit	Brazil	Focus group/ interview	10 Mothers, 7 HCPs	Premature: N/A cutoff	N/A	N/A	N/A	N/A	N/A	N/A	N/A	N/A

Author	Year	Title	Country	Study design	Sample size	Newborns Characteristics	KMC components	Onset of SSC	Stability criteria for KMC initiation	Hours per day of KMC provision*	# of days of KMC provision*	Discharge criteria for Kangarooed baby	Follow-up duration	Follow-up location
Sacks	2013	Neonatal care in the home in northern rural Honduras: a qualitative study of the role of traditional birth attendants	Honduras	Focus group/ interview	N/A TBA	N/A	N/A	N/A	N/A	N/A	N/A	N/A	N/A	N/A
Saeedi	2011	effect of kangaroo care method on the pain intensity of vaccination in newborns	Iran	RCT	60 Newborns	Full term, 2500-4000g	SSC	≥24h after birth, ≥30 min since last feeding, 2 min before vaccination	Apgar=7-11 at 1 min	0.5	1	N/A	N/A	N/A
Saeidi	2010	Kangaroo mother care for infantile colic: A randomized clinical trial	Iran	RCT	48 Newborns	Full term, >2500g	N/A	At first the parents received a dairy to report the initial assessment of infant for 2 days, then they were visited in clinic, and then they received another Diary for reporting KMC	Infants were term	≥2	7	N/A	N/A	N/A

Author	Year	Title	Country	Study design	Sample size	Newborns Characteristics	KMC components	Onset of SSC	Stability criteria for KMC initiation	Hours per day of KMC provision*	# of days of KMC provision*	Discharge criteria for Kangarooed baby	Follow-up duration	Follow-up location
								activities for 7 next days						
Saeidi	2011	Use of "kangaroo care" to alleviate the intensity of vaccination pain in newborns	Iran	RCT	60 Newborns	2500-4000g	SSC	30 min before vaccination	N/A	0.5	1	N/A	N/A	N/A
Sajedi	2007	The effect of Kangaroo Care on physiologic responses to pain of an intramuscular injection in neonates	Iran	RCT	100 Newborns	37-42 weeks, 2500-4000g	SSC	10 min before injection, when infant in a quiet and alert state	Apgar ≥8 at 1 min	≥0.21	1	N/A	N/A	N/A
Sakaki	2009	An investigation of the risk factors for infection with Methicillin-Resistant Staphylococcus Aureus among patients in a neonatal intensive care unit	Japan	Prospective Cohort	923 Newborns	All ages	SSC	N/A	N/A	≥1-2	N/A	N/A	N/A	N/A

Author	Year	Title	Country	Study design	Sample size	Newborns Characteristics	KMC components	Onset of SSC	Stability criteria for KMC initiation	Hours per day of KMC provision*	# of days of KMC provision*	Discharge criteria for Kangarooed baby	Follow-up duration	Follow-up location
Samra	2012	The effect of kangaroo mother care on the duration of phototherapy of infants readmitted for neonatal jaundice	Egypt	Intervention	50 Newborns	35-40 weeks	SSC	Once eligible: N/A definition	N/A	≥3	Every day until phototherapy stopped	N/A	N/A	N/A
Santos	2013	Maternal perception of the skin to skin contact with premature infants through the kangaroo position	Brazil	Focus group/ interview	12 Mothers	Premature and LBW:N/A cutoff,	SSC	N/A	N/A	N/A	N/A	N/A	N/A	N/A
Scher	2009	Neurophysiologic assessment of brain maturation after an 8-week trial of skin-to-skin contact on preterm infants	USA	Prospective Cohort	134 Newborns	28-32 weeks, >1000g, Full term	SSC	N/A	No encephalopathy or IVH > grade II or meningitis, or congenital brain malformations. No mothers' substance abuse.	1.5	5 days per week during 8 weeks	N/A	N/A	N/A
Schneider	2012	Cerebral motor function in very premature-at-birth adolescents: a brain stimulation exploration of kangaroo mother care effects	Colombia	RCT	48 Adolescents	Premature: N/A cutoff, Full term	SSC, DC, FU	Immediately after birth	Tolerating physical manipulation, able to suck and swallowing	N/A	N/A	Immediately after brief adaptation to KMC position	1 year	Health facility
Shu-guang	2012	Influence of Kangaroo care in improving milk intake and immune status for	China	RCT	80 Newborns	Premature: N/A cutoff	N/A	N/A	N/A	N/A	N/A	N/A	7, 14, and 28 days	N/A

Author	Year	Title	Country	Study design	Sample size	Newborns Characteristics	KMC components	Onset of SSC	Stability criteria for KMC initiation	Hours per day of KMC provision*	# of days of KMC provision*	Discharge criteria for Kangarooed baby	Follow-up duration	Follow-up location
		premature children												
Sikorová	2012	Verifying the benefits of kangaroo mother care - A pilot study	Czech Republic	RCT, Focus group/ interview	33 Newborns	<1800g	N/A	N/A	N/A	N/A	N/A	N/A	N/A	N/A
Silva	2010	Factors which influence weaning in preterm infant	Brazil	Retrospective cohort	89 Dyads	<37 weeks, <2500g	N/A	N/A	N/A	N/A	N/A	N/A	N/A	N/A
Silva	2008	Perceptions and neonatal care behavior of women in a Kangaroo-mother Care Program	Brazil	Focus group/ interview	5 Dyads	Premature: N/A cutoff, <1000-1550g	SSC	Once eligible: N/A definition	N/A	≤24	Depends mother's length of stay	N/A	N/A	N/A
Singh	2012	Utilization of postnatal care for newborns and its association with neonatal mortality in India: an analytical appraisal	India	Case control	145662 Newborns, 810204/ Mothers	All ages	N/A	N/A	N/A	N/A	N/A	N/A	N/A	N/A
Sloan	2008	Community-based kangaroo mother care to prevent neonatal and infant mortality: a randomized, controlled cluster trial	Bangladesh	Cluster RCT	4165 Newborns, 39888 Mothers	All ages	SSC	N/A	N/A	N/A	data available for first 2 days of life	N/A	N/A	N/A

Author	Year	Title	Country	Study design	Sample size	Newborns Characteristics	KMC components	Onset of SSC	Stability criteria for KMC initiation	Hours per day of KMC provision*	# of days of KMC provision*	Discharge criteria for Kangarooed baby	Follow-up duration	Follow-up location
Sloan	1994	Kangaroo mother method: randomized controlled trial of an alternative method of care for stabilized low-birth weight infants	Ecuador	RCT	300 Newborns	<2000g	SSC	Once tolerating food and weight stabilized	Temperature between 36.5-37.0 degrees C for 24h, tolerance of food, stable weight	N/A	N/A	N/A	1, 1.5, 2, 3, 4, 5, 6 mon. of life	Home and health facility
Smith	2003	Heart period variability of intubated very-low-birth-weight infants during incubator care and maternal holding	USA	Randomized cross-over	14 Newborns	24-30 weeks, 600-1500g, intubated	SSC	When the infant weighed at least 750g	≥Weight 750g	2	2	N/A	N/A	N/A
Solomons	2012	Knowledge and attitudes of nursing staff and mothers towards kangaroo mother care in the eastern sub-district of Cape Town	South Africa	Cross sectional	30 Mothers, 15 Nurses	<2500g	N/A	0.60 of mothers within 24 hours after birth	N/A	N/A	N/A	N/A	N/A	N/A
Stikes	2013	Applying the plan-do-study-act model to increase the use of kangaroo care	USA	Focus group/ interview	56 Nurses	N/A	SSC	N/A	N/A	N/A	N/A	N/A	N/A	N/A
Strand	2014	Kangaroo mother care in the neonatal intensive care unit: staff attitudes and beliefs and opportunities for par-	Sweden	Facility evaluation	126 Staff	N/A	N/A	N/A	N/A	N/A	N/A	N/A	N/A	N/A

Author	Year	Title	Country	Study design	Sample size	Newborns Characteristics	KMC components	Onset of SSC	Stability criteria for KMC initiation	Hours per day of KMC provision*	# of days of KMC provision*	Discharge criteria for Kangarooed baby	Follow-up duration	Follow-up location
		ents												
Subedi	2009	Kangaroo mother care for low birth weight babies: A prospective observational study	Nepal	Prospective Cohort	60 Newborns	1200-2000g	SSC, EBF, FU	Once eligible: N/A definition	N/A	N/A	N/A	Weight gain ≥10gm/kg/day for ≥3 consecutive days, stable and maintaining vitals, no sepsis, mothers confident caring babies at home	Weekly until 2500g	N/A
Suman	2008	Kangaroo mother care for low birth weight infants: a randomized controlled trial	India	RCT	206 Newborns	<2000g	SSC, EBF, FU	As soon as the baby was stable	no critically ill requiring ventilator or ionotropic support, no chromosomal or life threatening congenital anomalies, no requiring transfer, no critically ill mothers	as long as possible during the day and night	N/A	Weight gain of 10-15 g/kg/d for 3 consecutive days, feeding well, maintaining temperature mother confident of caring baby	Weekly until PMA of 40 weeks in preterm or until a weight of 2500 g in term SGA babies	Health facility
Suzuki	2013	Effect of early skin-to-skin contact on breast-feeding	Japan	Prospective cohort	470 Dyads	N/A	SSC, EBF, FU	Immediately after birth	N/A	1-1.5	1	N/A	1 mon.PGA	Home

Author	Year	Title	Country	Study design	Sample size	Newborns Characteristics	KMC components	Onset of SSC	Stability criteria for KMC initiation	Hours per day of KMC provision*	# of days of KMC provision*	Discharge criteria for Kangarooed baby	Follow-up duration	Follow-up location
Sylla	2011	Towards better care for preterm infants in Bamako, Mali	Mali	Prospective cohort, no control	480 Newborns	Premature: N/A cutoff, <2000g	SSC, DC, FU	Once eligible: N/A definition	Clinically stable (autonomous breathing)	N/A	N/A	As soon as weight gain and parents' independent	Initially twice a week, then once a week until term (40wks), then mon.till 6mo, then every 2 mon. until 12-18 mon.	Health facility
Takahashi	2011	Comparison of salivary cortisol, heart rate, and oxygen saturation between early skin-to-skin contact with different initiation and duration times in healthy, full-term infants	Japan	Intervention	108 Newborns	Full term	SSC	Immediately (≤5min) after birth in one group, after initial newborn exam (>5min after birth) in other group	N/A	N/A	N/A	N/A	N/A	N/A
Tallandini	2006	Kangaroo Mother Care and mother-premature infant dyadic interaction	Italy	Intervention	40 Dyads	<1800g	SSC	N/A	N/A	≥1	N/A	Sufficient physiological stability	N/A	N/A
Tenório	2010	Evaluation of physiological parameters in preterm newborns with low weight before and	Brazil	Pre-post	24 Newborns	<24-36 weeks, <2500g	N/A	N/A	N/A	0.33	3	N/A	N/A	N/A

Author	Year	Title	Country	Study design	Sample size	Newborns Characteristics	KMC components	Onset of SSC	Stability criteria for KMC initiation	Hours per day of KMC provision*	# of days of KMC provision*	Discharge criteria for Kangarooed baby	Follow-up duration	Follow-up location
		after application of the kangaroo mother care												
Tessier	1998	Kangaroo mother care and the bonding hypothesis	Colombia	RCT	488 Newborns	<2001g	SSC, DC, FU	Once adapted to extra-uterine life and able to breast-feed	Adapted to extra-uterine life	N/A	N/A	Weight gain ≥20 g /d, ≥ 1700 g	Weekly until term (40 weeks GA)	Health facility
Tessier	2009	Kangaroo Mother Care, home environment and father involvement in the first year of life: a randomized controlled study	Colombia	RCT	338 Dyads	2001g	SSC, DC, FU	Premature infant adapted to extra-uterine life and able to breast-fed	The same as SSC onset criteria	N/A	N/A	Premature infant adapted to extra-uterine life and able to breastfeed	Original RCT included weekly visits until 40 wks GA, then interview/evaluation at 9-12 mon.	Health facility

Author	Year	Title	Country	Study design	Sample size	Newborns Characteristics	KMC components	Onset of SSC	Stability criteria for KMC initiation	Hours per day of KMC provision*	# of days of KMC provision*	Discharge criteria for Kangarooed baby	Follow-up duration	Follow-up location
Tessier	2003	Kangaroo Mother Care: A method for protecting high-risk low-birth-weight and premature infants against developmental delay	Colombia	RCT	431 Dyads	Premature: N/A cutoff, ≤1800g	SSC, DC, FU	Premature infant has adapted to extra-uterine life and is able to breast-feed	The same as SSC onset criteria	N/A	N/A	The same as SSC onset criteria	followed daily until gaining ≥20g/day then weekly until term (40wks GA), then interview/evaluation at 12 mon.	Health facility
Thukral	2012	Early skin-to-skin contact and breast-feeding behavior in term neonates: A randomized controlled trial	India	RCT	41 Dyads	Full term	SSC, EBF, FU	Immediately after birth	N/A	2	1	N/A	N/A	Health facility
Toma	2003	Kangaroo Mother Care: the role of health care services and family networks in a successful program	Brazil	Focus group/ interview	14 Mothers, 7 Fathers	Premature: N/A cutoff, 1150-2300g	N/A	Ranged from 3 to 39 days of life	N/A	N/A	N/A	Clinical stability	Until get 2500 g	Home and health facility
Toma	2007	Maternal perception of low birth weight babies before and following the implementation of the Kangaroo Mother Care in a public hospital, in the city of São Paulo, Brazil	Brazil	Focus group/ interview	41 Mothers	<2000g	N/A	On mean 18 days	N/A	N/A	N/A	Knowledge with breast-feeding, gain weight	15 days after the discharge, till 6 mon. after discharge	Health facility

Author	Year	Title	Country	Study design	Sample size	Newborns Characteristics	KMC components	Onset of SSC	Stability criteria for KMC initiation	Hours per day of KMC provision*	# of days of KMC provision*	Discharge criteria for Kangarooed baby	Follow-up duration	Follow-up location
Tornhage	1999	First week kangaroo care in sick very preterm infants	Sweden	Pre-post	17 Newborns	24-30 weeks	N/A	N/A	When infant can tolerate handling	≥1	N/A	N/A	N/A	N/A
Tuoni	2012	Kangaroo Mother Care: four years of experience in very low birth weight and preterm infants	Italy	Retrospective cohort	213 Newborns	Premature: N/A cutoff, 1500g	SSC	Once eligible: N/A definition	Stability of vital function – nor more definition	≥1	N/A	N/A	N/A	N/A
Undefined author** Save the Children	2011	Scaling Up Kangaroo Mother Care, Report of Country Survey Findings	Ethiopia, Malawi, Mali, Mozambique, Nigeria, Tanzania, Uganda, Bolivia, Indonesia, Nepal, Vietnam	Facility evaluation	12 Countries	N/A	N/A	N/A	N/A	N/A	N/A	N/A	N/A	N/A
Van Zanten	2007	The kangaroo method is safe for premature infants under 30 weeks of gestation during ventilatory support	Netherlands	Pre-post	34 Newborns	25-30 weeks	N/A	N/A	N/A	≥1	N/A	N/A	N/A	N/A
Vargas García	2012	Most frequent diseases in newborns of the kangaroo program of Clínica Universitaria Bolivariana in Medellin from June to December 2009	Bolivia	Chart review	86 Newborns	<37 weeks, <2500g	N/A	N/A	N/A	N/A	N/A	N/A	N/A	N/A

Author	Year	Title	Country	Study design	Sample size	Newborns Characteristics	KMC components	Onset of SSC	Stability criteria for KMC initiation	Hours per day of KMC provision*	# of days of KMC provision*	Discharge criteria for Kangarooed baby	Follow-up duration	Follow-up location
Vesel	2013	Promoting skin-to-skin care for low birth weight babies: findings from the Ghana Newhints cluster-randomized trial	Ghana	Cluster RCT	15615 Newborns, 98 Zones	All ages	SSC	N/A	N/A	N/A	N/A	N/A	9 weekly visit in last 6 mon. of study	Home
Wahlberg	1992	A retrospective, comparative study using the kangaroo method as a complement to the standard incubator care	Sweden	Retrospective cohort	66 Dyads	Premature: N/A cutoff	SSC	N/A	N/A	N/A	N/A	N/A	N/A	N/A
Waiswa	2010	'I never thought that this baby would survive; I thought that it would die any time': perceptions and care for preterm babies in eastern Uganda	Uganda	Focus group/ interview	30 Mix of HCWs and mothers, 16 Facilities	Premature: N/A cutoff	N/A	N/A	N/A	N/A	N/A	N/A	N/A	N/A
Wallin	2005	Staff experiences in implementing guidelines for Kangaroo Mother Care - A qualitative study	Sweden	RCT, Focus group/ interview	45 Staff at 4 facilities	N/A	N/A	N/A	N/A	N/A	N/A	N/A	N/A	N/A

Author	Year	Title	Country	Study design	Sample size	Newborns Characteristics	KMC components	Onset of SSC	Stability criteria for KMC initiation	Hours per day of KMC provision*	# of days of KMC provision*	Discharge criteria for Kangarooed baby	Follow-up duration	Follow-up location
Weller	2002	Longitudinal assessment of pituitary-thyroid axis and adrenal function in preterm infants raised by 'kangaroo mother care'	Colombia	RCT	87 Newborns	<37 weeks, <2001g	SSC, DC, FU	N/A	N/A	N/A	N/A	As soon as no infections, regardless of weight or GA	Daily until weight gain ≥20g/day	Health facility
Whitelaw	1988	Skin to skin contact for very low birth weight infants and their mothers	UK	RCT	71 Newborns, 63 Mothers	<1500g	SSC	Once eligible: N/A definition	Unassisted breathing if infant in the trial became unwell, trial was discontinued until infant stable again	N/A	N/A	N/A	6 mon. for all infants, if still breastfeeding, lactation duration assessed at 9 and 12 mon.	Health facility
White-Traut	2012	Frequency of Premature Infant Engagement and Disengagement Behaviors During Two Maternally Administered Interventions	USA	RCT	26 Dyads	Premature: N/A cutoff	SSC	N/A	≥1000 grams and assessed as clinically stable	1.5	3 hours/ week	N/A	N/A	N/A
Wieland	1995	Skin-to-skin care with 39 preterm infants	Germany	Prospective Cohort	39 Newborns	25-32 weeks, 740-2125g	N/A	10 days post natal age (4-40 day)	N/A	N/A	N/A	N/A	N/A	N/A
Wobi**	2010	Report on the Kangaroo Mother Care Project at Komfo Anokye Teaching Hospital in Kumasi, Ghana	Ghana	Facility evaluation	2 Facilities	N/A	N/A	N/A	N/A	N/A	N/A	N/A	N/A	N/A

Author	Year	Title	Country	Study design	Sample size	Newborns Characteristics	KMC components	Onset of SSC	Stability criteria for KMC initiation	Hours per day of KMC provision*	# of days of KMC provision*	Discharge criteria for Kangarooed baby	Follow-up duration	Follow-up location
Worku	2005	Kangaroo mother care: a randomized controlled trial on effectiveness of early kangaroo mother care for the low birth weight infants in Addis Ababa, Ethiopia	Ethiopia	RCT	123 Newborns	<2000g	SSC, EBF	Immediately after birth	No congenital anomalies; healthy mother	N/A	N/A	Stable temperature, stabilized cardiovascular status, ability to suck, good general condition	N/A	N/A
Xuefen	2013	Effect of Kangaroo care on diminishing neonatal pain during heel lancing	China	RCT	60 Newborns	37-42 weeks, 2500-4000g	N/A	Once eligible: N/A definition	N/A	N/A	N/A	N/A	N/A	N/A

bpm: beat per minute; CA: corrected Age; CHW: community health workers; DC: early discharge from facility; D/W: days/week; EBF: exclusively or near exclusively breast feeding; FU: follow up after discharge;
HCP: health care professionals; HCW: health care worker; HR: heart rate; IV: intravenous; IVH: intra ventricular hemorrhage; KMC: kangaroo mother care; LBW: low birth weight; min: minutes; mon.: months;
N/A: undefined or not available; NG tube: naso-gastric tube; PCA: post cenceptional age; PGA: post gestational age; PMA: post menstrual age; RR: respiratory rate; SSC:skin-to-skin contact;TBA: traditional birth attendance
* Number of hours per day and number of days of KMC provision were amount of KMC provision promoted by researchers not truly observed.
** Program report
